# Supplementary material for: Enhanced Generation of Integration-free iPSCs from Human Adult Peripheral Blood Mononuclear Cells with an Optimal Combination of Episomal Vectors
Source: Stem Cell Reports. 2016 May 5;6(6):873–84. doi: 10.1016/j.stemcr.2016.04.005 (PMC4911493; doi:10.1016/j.stemcr.2016.04.005)
Supplement: Document S1. Supplemental Experimental Procedures, Figures S1–S3, and Table S1 [file mmc1.pdf]

Stem Cell Reports, Volume *6*

Supplemental Information

Enhanced Generation of Integration-free iPSCs from Human Adult Peripheral Blood Mononuclear Cells with an Optimal Combination of Episomal Vectors

Wei Wen, Jian-Ping Zhang, Jing Xu, Ruijun Jeanna Su, Amanda Neises, Guang-Zhen Ji, Weiping Yuan, Tao Cheng, and Xiao-Bing Zhang

Stem Cell Reports, Volume 6

Supplemental Information

Enhanced Generation of Integration-free iPS Cells from Human Adult Peripheral Blood Mononuclear Cells with an Optimal Combination of Episomal Vectors

Wei Wen[1*], Jian-Ping Zhang[1*], Jing Xu[1*], Ruijun Jeanna Su[2], Amanda Neises[2], Guang-Zhen Ji[1], Weiping Yuan[1], Tao Cheng[1], Xiao-Bing Zhang[1,2]

[1] State Key Laboratory of Experimental Hematology, Institute of Hematology and Blood Disease Hospital, Chinese Academy of Medical Sciences and Peking Union Medical College, Tianjin 300020,China

[2] Department of Medicine, Loma Linda University, Loma Linda, CA 92350, USA

*These authors contributed equally to this work.

SUPPLEMENTAL FIGURES AND FIGURE LEGENDS

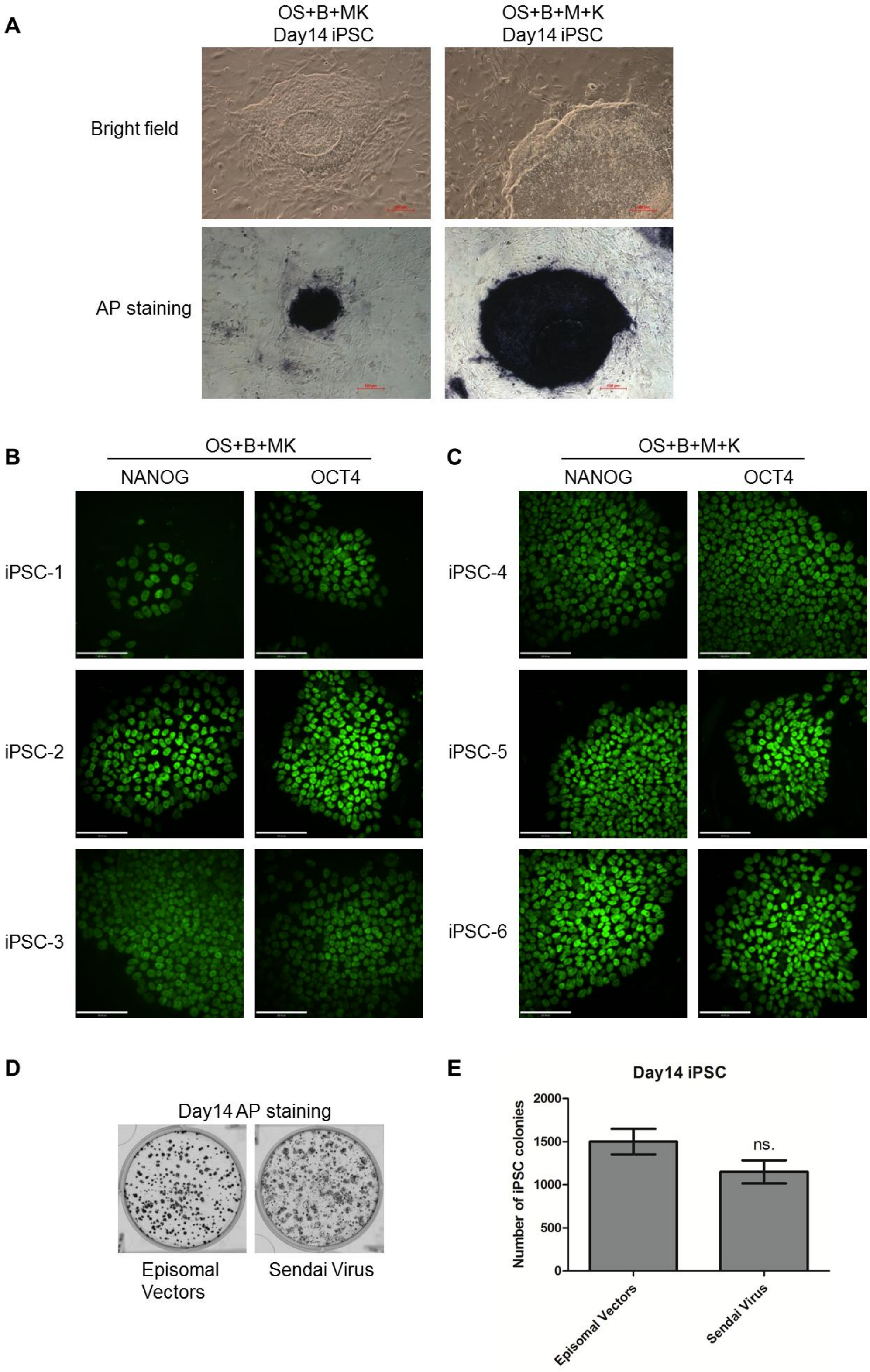

Figure S1 Related to Figure 1.

(A) Representative images of morphology and AP staining of iPSC colonies.

(B) and (C) Fluorescent images of iPSC colonies expressing OCT4 and NANOG. iPSCs were generated by nucleofection with OS+B+MK (B) and OS+B+M+K (C). Scale bar, 100 μm.

(D) Representative pictures of AP staining of iPSCs generated from $1x10^5$ PB MNCs with the EV vs. Sendai virus reprogramming system.

(E) Numbers of iPSC colonies from $1x10^6$ PB MNCs (Mean ± SEM, n = 3 independent experiments).

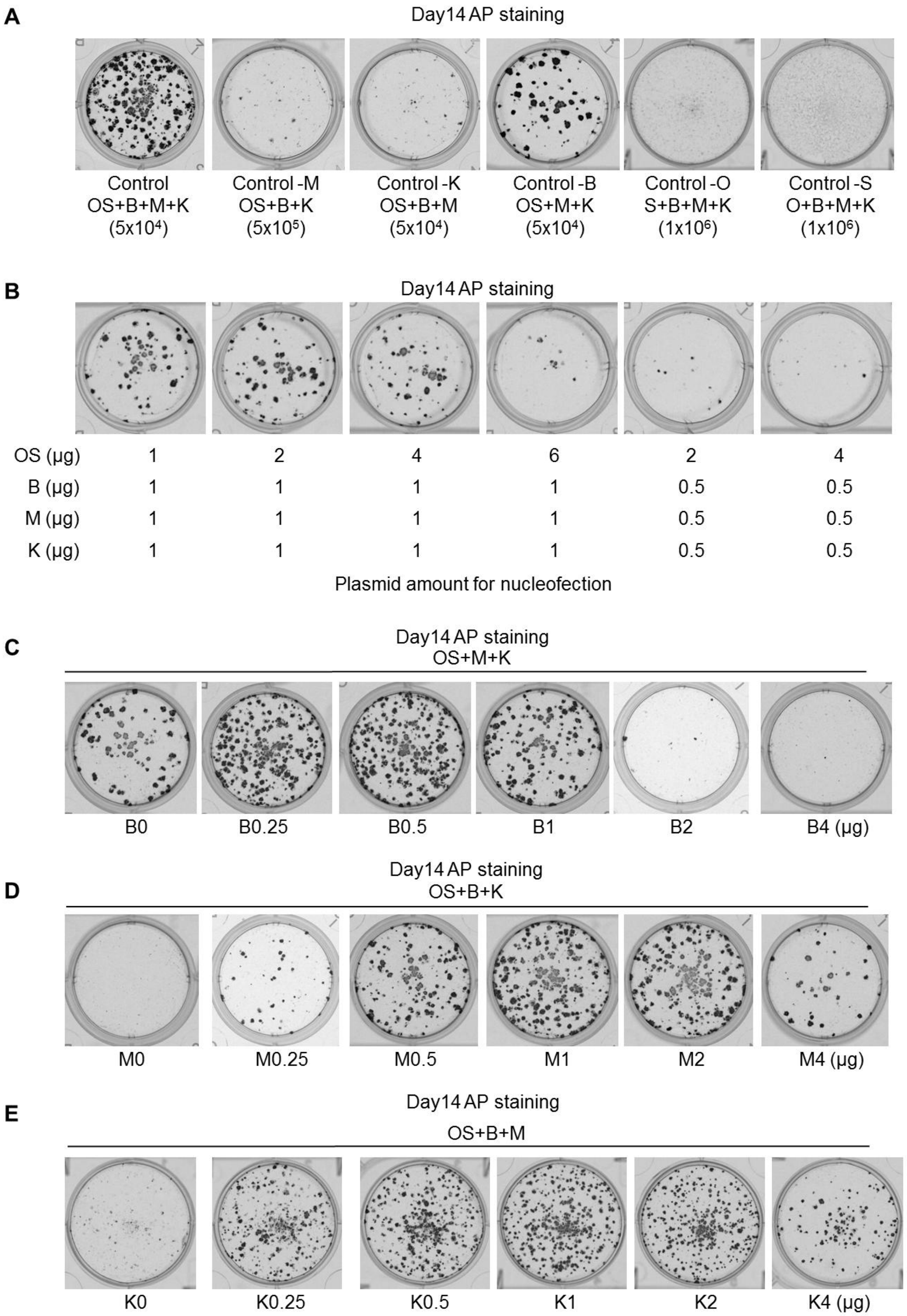
A
Day14 AP staining
Control
OS+B+M+K
(5x10⁴)
Control -M
OS+B+K
(5x10⁵)
Control -K
OS+B+M
(5x10⁴)
Control -B
OS+M+K
(5x10⁴)
Control -O
S+B+M+K
(1x10⁶)
Control -S
O+B+M+K
(1x10⁶)
B
Day14 AP staining
OS (µg) 1 2 4 6 2 4
B (µg) 1 1 1 1 0.5 0.5
M (µg) 1 1 1 1 0.5 0.5
K (µg) 1 1 1 1 0.5 0.5
Plasmid amount for nucleofection
C
Day14 AP staining
OS+M+K
B0
B0.25
B0.5
B1
B2
B4 (µg)
D
Day14 AP staining
OS+B+K
M0
M0.25
M0.5
M1
M2
M4 (µg)
E
Day14 AP staining
OS+B+M
K0
K0.25
K0.5
K1
K2
K4 (µg)

Figure S2 Related to Figure 2.

(A) Representative images of AP staining at day 14 post-transfection. Omission of one factor was tested in each condition. Note that the input cell numbers are different.

(B) Representative images of AP staining at day 14 post-transfection. $5x10^4$ PB MNCs were seeded in each well after nucleofection with indicated amount of each plasmid.

(C) Dosage effects of BCL-XL. Shown are representative images of AP staining of iPSCs 14 days after transfection with 2µg OS, 1µg M, 1µg K together with different amount of BCL-XL. $5x10^4$ PB MNCs were seeded in each well after nucleofection.

(D) Dosage effects of MYC. Shown are representative images of AP staining of iPSCs 14 days after transfection with 2µg OS, 1µg B, 1µg K together with different amount of MYC. $5x10^4$ PB MNCs were seeded in each well after nucleofection.

(E) Dosage effects of KLF4. Shown are representative images of AP staining of iPSCs 14 days after transfection with 2µg OS, 1µg B, 1µg M together with different amount of KLF4. $5x10^4$ PB MNCs were seeded in each well after nucleofection.

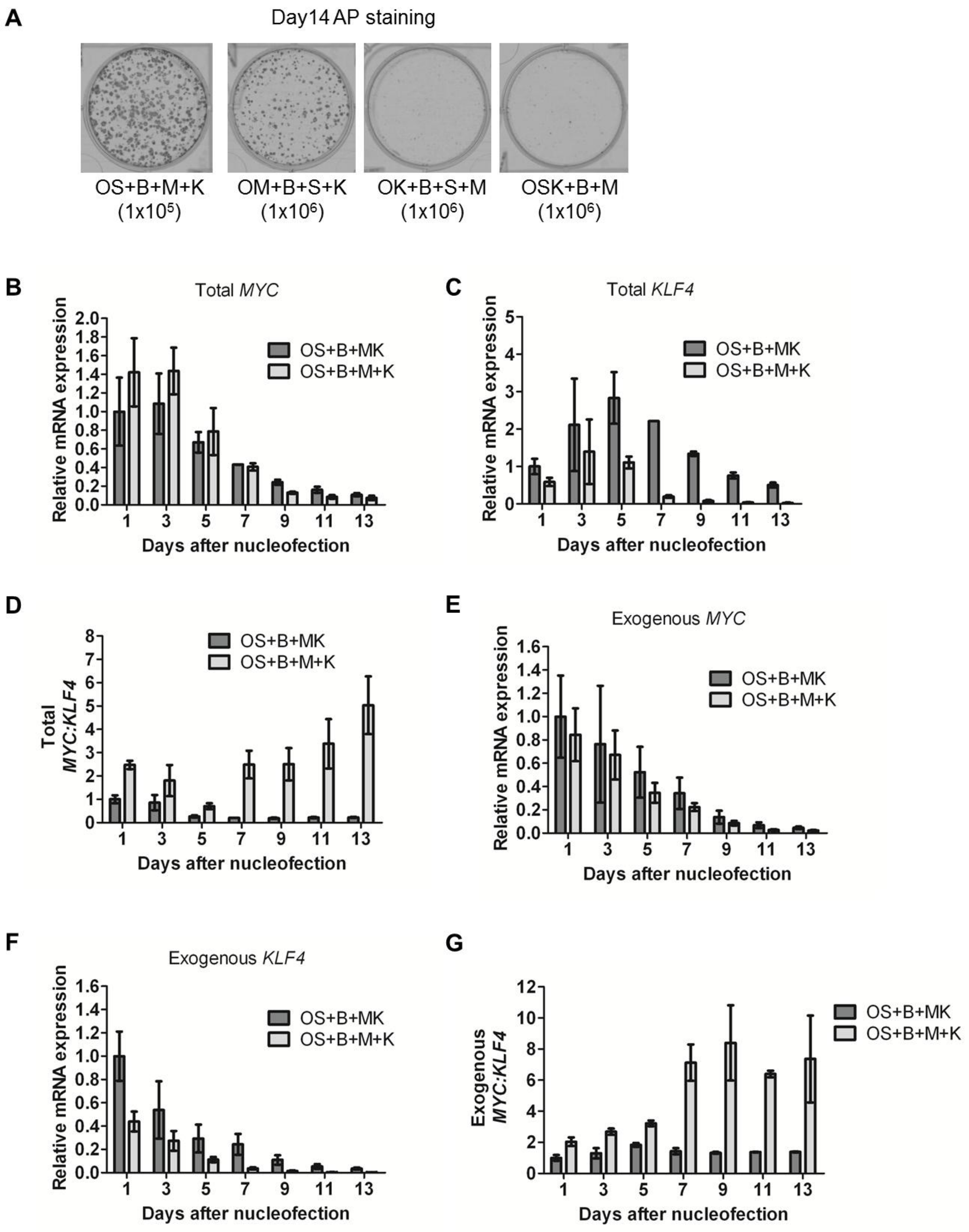
A
Day14 AP staining
OS+B+M+K (1x10⁵)
OM+B+S+K (1x10⁶)
OK+B+S+M (1x10⁶)
OSK+B+M (1x10⁶)
B
Total MYC
Relative mRNA expression
Days after nucleofection
OS+B+MK
OS+B+M+K
C
Total KLF4
Relative mRNA expression
Days after nucleofection
OS+B+MK
OS+B+M+K
D
Total MYC:KLF4
Days after nucleofection
OS+B+MK
OS+B+M+K
E
Exogenous MYC
Relative mRNA expression
Days after nucleofection
OS+B+MK
OS+B+M+K
F
Exogenous KLF4
Relative mRNA expression
Days after nucleofection
OS+B+MK
OS+B+M+K
G
Exogenous MYC:KLF4
Days after nucleofection
OS+B+MK
OS+B+M+K

Figure S3 Related to Figure 3.

(A) Representative images of AP staining of iPSC induction with different combinations of plasmids. Note that input cell numbers are different.

(B) Relative total *MYC* mRNA expression levels during reprogramming with OS+B+MK vs. OS+B+M+K. The expression levels are normalized to OS+B+MK at day 1 (Mean ± SEM, n = 3 independent experiments).

(C) Relative total *KLF4* mRNA expression levels during reprogramming in OS+B+MK vs. OS+B+M+K. The expression levels are normalized to OS+B+MK at day 1 (Mean ± SEM, n = 3 independent experiments).

(D) Relative ratios of total *MYC* to total *KLF4* during reprogramming in OS+B+MK vs. OS+B+M+K (Mean ± SEM, n = 3 independent experiments). The ratios are normalized to OS+B+MK at day 1.

(E) Relative expression of exogenous *MYC* at indicated time points after transfection with OS+B+MK vs. OS+B+M+K vectors. The expression levels are normalized to OS+B+MK at day 1 (Mean ± SEM, n = 3 independent experiments).

(F) Relative expression of exogenous *KLF4* at indicated time points after transfection with OS+B+MK vs. OS+B+M+K vectors. The expression levels are normalized to OS+B+MK at day 1 (Mean ± SEM, n = 3 independent experiments).

(G) Relative ratios of exogenous *MYC* to exogenous *KLF4* during reprogramming in OS+B+MK vs. OS+B+M+K (Mean ± SEM, n = 3 independent experiments). The ratios are normalized to OS+B+MK at day 1.

Supplemental Table

Table S1 Primer sequences used in the study.

Gene	Direction	Sequence
Total MYC	Forward	ACTCTGAGGAGGAACAAGAA
	Reverse	TGGAGACGTGGCACCTCTT
Exogenous MYC	Forward	TCCTCCGACAGACTGAGTC
	Reverse	CCTCCTCGTCGCAGTAGAAA
Total KLF4	Forward	TCTCAAGGCACACCTGCGAA
	Reverse	TAGTGCCTGGTCAGTTCATC
Exogenous KLF4	Forward	GAAATTCGCCCGCTCAGATG
	Reverse	TCATTTGCTGCCAGATCCTC
EBNA1	Forward	TTTAATACGATTGAGGGCGTCT
	Reverse	GGTTTTGAAGGATGCGATTAAG
Wpre	Forward	GGTTTAAACGCGTCGACAAT
	Reverse	GTTGCGTCAGCAAACACAGT
GAPDH	Forward	GAGTCCACTGGCGTCTTC
	Reverse	GACTGTGGTCATGAGTCCTTC

SUPPLEMENTAL EXPERIMENTAL PROCEDURES

AP Staining

The reprogramming efficiency was determined by counting AP-positive colonies at day 14. Cells were first fixed in 4% paraformaldehyde and subsequently stained for AP activities with an Alkaline Phosphatase Detection Kit (SiDanSai, China, 1102-100), following manufacturer's instructions. The colony numbers were scored by IQTL7.0 software or manually under a microscope.

Flow Cytometry

Cells were harvested with Accutase, followed by staining for 20 min at room temperature with PE-conjugated anti-TRA-1-60 (BioLegend; 330610), or eFluor 570-conjugated anti-SSEA4 (eBioscience; 41-8843). The cells were also stained with isotype antibody (eBioscience, 11-4011) for the negative control. After one wash with 2 ml PBS, cells were resuspend in 300 µl PBS for FACS analysis. Data were acquired using a BD LSR Fortessa cell analyzer.

Genomic DNA Extraction

Genomic DNA was extracted from cells using a Genomic DNA Extraction Kit (TIANGEN, DP304-02), following the manufacturers protocol.

Examination of residual plasmid by PCR

To determine the total copies of EV plasmid in iPSC lines, the Wpre and EBNA primers targeting the EV backbone were used (Table S1). PCR was performed using Phusion® High-Fidelity PCR Master Mix with HF Buffer Kit (BioLabs, M0531S). Briefly, 9 µl PCR grade water including 100 ng genomic DNA and 1 µl specific primers (10 µM each of forward and reward primers, Table S1) were added into 10 µl PCR Master Mix. The reaction mixture was incubated for 30 s at 98°C; followed by 10 s at 98°C, 30 s at 60°C and 30 s at 72°C for 35 cycles; and final extension for 5 min at 72°C. Genomic DNA from untransfected PB MNCs were used as a negative control. To quantify the EV copy number, 1.6 pg OS plasmid was mixed with 1 µg gDNA from untransfected PB MNCs to mimic cells with one copy of EV plasmid per cell.

RNA Extraction and cDNA Synthesis

Total RNA was extracted from cells using an RNeasy Mini Kit (Qiagen, 74104). cDNAs were synthesized with M-MLV Reverse Transcriptase (Invitrogen, 28025) and stored at -20°C until use.

Real-time PCR

Real-time PCR was performed using a FastStart Universal SYBR Green Master (Roche) on a Step One 7500 (Applied Biosystems). Briefly, 5 µl cDNA (1-10 ng) or 100 ng genomic DNA was added into 10 µl the SYBR Green Master together with 5 µl primers (2 µM each of forward and reward primers, Table S1). The reaction mixture was incubated for 10 min at 95°C, followed by 15 s at 95°C and 1 min at 60°C for 40 cycles. Data were analyzed using the Applied Biosystems Step One Software v2.2. Amplification of *GAPDH* was also conducted to control the quantity of loaded cDNA or gDNA in each reaction.

Confocal Imaging

iPSC lines were seeded onto feeder cells or Matrigel coated chamber slides. Cells were fixed in 4% paraformaldehyde for 30 min, followed by permeabilization treatment with 0.1% Triton X-100 in PBS for 30 min. After washing with PBS, cells were treated with blocking solution (5% goat serum in PBS, v:v) for 1 h, followed by overnight at 4°C with diluted primary antibody in blocking reagent. After two washes with permeabilization buffer, cells were incubated with a fluorophore-conjugated secondary antibody in blocking reagent for 2 h at room temperature. DAPI was used to stain the nucleus before imaging. Images were captured with a PE UltraVIEW confocal microscope.

Teratoma Assay

Approximately $1x10^6$ iPSCs after more than 5 passages in culture were harvested with Accutase and resuspended in 200 µl DMEM/F12 diluted (1:1) Matrigel solution (BD) and subcutaneously injected into the rear haunch of NOD/SCID immunodeficient mice. At 2 months after implantation, teratomas were dissected and fixed in 10% formalin. After microsectioning and staining with hematoxylin and eosin (H & E), samples were analyzed.

Karyotyping

iPSCs were cultured in Matrigel-treated 6-well plates. After reaching a 60%-70% confluence, cells were incubated with colchicines (0.2 µg/ml) for 3 to 4 hours, followed by treating with 0.05 mM EDTA for 3 min at 37°C. Cells were harvested with Accutase and single cell suspension was treated with 0.4% KCl for 25 min at 37°C. Cells were then fixed with 3-5 drops of fixation buffer (Methanol: Acetic Acid (v:v)= 3:1) for 5 min at room temperature. Finally, cells were centrifuged at 200 g for 8 min and treated with cold fixing buffer for 20 min at room temperature twice. The cell suspension was dropped onto cold clear slides followed by overnight incubation at 60°C in an incubator. The chromosomes were digested by 0.025% trypsin (Invitrogen) and stained with Giemsa stain solution (Sigma, 32884). The karyotyping analysis was performed using MetaSystem (Zeiss Imager Z2).
