# Supplementary material for: Enhanced Generation of Integration-free iPSCs from Human Adult Peripheral Blood Mononuclear Cells with an Optimal Combination of Episomal Vectors
Source: Stem Cell Reports. 2016 May 5;6(6):873–84. doi: 10.1016/j.stemcr.2016.04.005 (PMC4911493; doi:10.1016/j.stemcr.2016.04.005)
Supplement: Document S2. Article plus Supplemental Information [file mmc2.pdf]

Stem Cell Reports
Article
OPEN ACCESS

Enhanced Generation of Integration-free iPSCs from Human Adult Peripheral Blood Mononuclear Cells with an Optimal Combination of Episomal Vectors

Wei Wen,[1,3] Jian-Ping Zhang,[1,3] Jing Xu,[1,3] Ruijun Jeanna Su,[2] Amanda Neises,[2] Guang-Zhen Ji,[1] Weiping Yuan,[1] Tao Cheng,[1,*] and Xiao-Bing Zhang[1,2,*]
[1]State Key Laboratory of Experimental Hematology, Institute of Hematology and Blood Diseases Hospital, Collaborative Innovation Center for Cancer Medicine, Chinese Academy of Medical Sciences and Peking Union Medical College, Tianjin 300020, China
[2]Division of Regenerative Medicine MC1528B, Department of Medicine, Loma Linda University, 11234 Anderson Street, Loma Linda, CA 92350, USA
[3]Co-first author
*Correspondence: chengtao@ihcams.ac.cn (T.C.), xzhang@llu.edu (X.-B.Z.)
http://dx.doi.org/10.1016/j.stemcr.2016.04.005

SUMMARY

We previously reported the generation of integration-free induced pluripotent stem cells from adult peripheral blood (PB) with an improved episomal vector (EV) system, which uses the spleen focus-forming virus U3 promoter and an extra factor BCL-XL (B). Here we show an ~100-fold increase in efficiency by optimizing the vector combination. The two most critical factors are: (1) equimolar expression of OCT4 (O) and SOX2 (S), by using a 2A linker; (2) a higher and gradual increase in the MYC (M) to KLF4 (K) ratio during the course of reprogramming, by using two individual vectors to express M and K instead of one. The combination of EV plasmids (OS + M + K + B) is comparable with Sendai virus in reprogramming efficiency but at a fraction of the cost. The generated iPSCs are indistinguishable from those from our previous approach in pluripotency and phenotype. This improvement lays the foundation for broad applications of episomal vectors in PB reprogramming.

INTRODUCTION

One decade ago, Takahashi and Yamanaka (2006) made a stunning discovery that mouse somatic cells can be reprogrammed into a pluripotent state after forced expression of defined factors composed of OCT4 (also known as POU5F1), SOX2, KLF4, and MYC (also termed c-MYC). The finding in mouse cells was soon reproduced with human fibroblasts (Takahashi et al., 2007; Yu et al., 2007). This breakthrough has changed the landscape of personalized cell therapy, disease modeling, and drug screening.

Fibroblasts are the widely used cellular source for many reprogramming studies reported in the last decade but with noticeable limitations (Zhang, 2013). A major drawback is that the derivation of a sufficient amount of fibroblasts for reprogramming requires a lengthy 2–3 weeks of in vitro culture. Human fibroblasts are often obtained by skin biopsy, which is an invasive and non-sterile procedure. Of more concern, skin cells bear more mutations due to environmental insults than cells from inside the body (Abyzov et al., 2012).

In contrast to dermal fibroblasts, peripheral blood (PB) has been widely used in medical diagnostics and is the most accessible resource to procure large quantities of cells. Compared with human fibroblasts, PB can be obtained from freshly drawn samples or existing blood stocks. After drawing blood, gradient centrifugation separates red blood cells and granulocytes from mononuclear cells (MNCs) with lower density (Zhang, 2013). The original protocol using retroviral vectors expressing Yamanaka factors (OCT4, SOX2, KLF4, and MYC) has been found to be successful in many cell types, including hematopoietic cells (Aasen et al., 2008; Broxmeyer et al., 2011; Loh et al., 2009; Mali et al., 2008, 2010; Park et al., 2008). Reprogramming of T cells, a major subpopulation of MNCs, into pluripotency has been achieved by many laboratories using different approaches (Loh et al., 2010; Okita et al., 2013; Staerk et al., 2010) and T cell reprogramming has the potential to rejuvenate aged T cells for immunotherapy (Nishimura et al., 2013; Wakao et al., 2013). However, induced pluripotent stem cells (iPSCs) from non-lymphoid cells may be more useful, since mature T cells harbor a single T cell receptor (TCR) after somatic recombination and are unable to regenerate the T cell repertoire with unlimited possibilities. In contrast to mature T cells, hematopoietic progenitors contain an intact genome and are readily reprogrammable after ex vivo expansion in conditions that favor the proliferation of myeloid cells or erythroid cells (Agu et al., 2015; Chou et al., 2011, 2015; Diecke et al., 2015; Dowey et al., 2012; Hu et al., 2011; Liu et al., 2014; Loh et al., 2009; Mack et al., 2011; Meng et al., 2012; Merling et al., 2013; Okita et al., 2013).

For cell replacement therapies, the use of integration-free iPSCs that bear no exogenous genetic elements is required. We and other groups have demonstrated that episomal vectors (EV) are capable of reprogramming human somatic cells, including MNCs, into integration-free iPSCs (Chou et al., 2011, 2015; Dowey et al., 2012; Meraviglia et al., 2015; Okita et al., 2013; Su et al., 2013a, 2016; Yu et al., 2009, 2011). The most commonly used EV is a plasmid incorporated with two elements from the Epstein-Barr (EB) virus, origin of viral replication (oriP) and EB nuclear

© 2016 The Authors
This is an open access article under the CC BY license (http://creativecommons.org/licenses/by/4.0/).

antigen 1 (EBNA1) (Dorigo et al., 2004). One transfection of the EV is sufficient for iPSC generation due to oriP/EBNA1-mediated plasmid retention in mammalian cells, while a gradual loss of EV during each cell division eventually renders the iPSC lines to become void of ectopic factors (Chou et al., 2011; Okita et al., 2013). However, EV-mediated reprogramming was very inefficient, thus potentially risky factors such as SV40 large T antigen and p53 shRNA were used in some studies to achieve appreciable efficiency (Okita et al., 2011; Yu et al., 2009). For reprogramming of human PB MNCs, the success rate was frustratingly low without SV40 large T antigen and p53 suppression (Chou et al., 2011; Dowey et al., 2012). With the use of spleen focus-forming virus U3 (SFFV), a strong promoter in hematopoietic cells, and an additional pro-survival factor BCL-XL, the reprogramming efficiency of PB MNCs was increased by 10- to 100-fold (Meng et al., 2012; Su et al., 2013a, 2016).

In many studies, the reprogramming factors were delivered individually using monocistronic vectors. However, due to differences in vector uptake, expression levels of each gene in each cell are highly variable (Lo et al., 2015). Since the ratio between the factors is one of most critical factors for successful reprogramming (Carey et al., 2011; Kim et al., 2015; Papapetrou et al., 2009), the optimal stoichiometry of the reprogramming factors enhances reprogramming efficiency. To achieve equimolar expression of multiple proteins, genes can be linked with self-cleaving 2A-like sequences of CHYSEL polypeptides, which are used by RNA viruses to separate multiple viral genes to be translated (de Felipe et al., 2006). In this system, cleavage occurs through ribosomal skipping during translation, resulting in the release of the upstream protein while translation of the downstream mRNA continues. Commonly used 2A peptides in research are from foot-and-mouth disease virus (F2A), equine rhinitis A virus (E2A), porcine teschovirus-1 (P2A), and *Thosea asigna* virus (T2A) (Yang et al., 2008). As our OS vector linked with E2A can efficiently reprogram hematopoietic cells (Meng et al., 2012; Su et al., 2013a, 2016), we use E2A to link two or more genes to ensure the equimolar expression of several genes in this study.

In our previous study, using three EV plasmids encoding OCT4-E2A-SOX2 (OS), BCL-XL (B), and MYC-E2A-KLF4 (MK) (OS + B + MK), we generated 20–30 integration-free iPSCs from 1 × 10^6 cultured MNCs or ~1 ml of PB (Su et al., 2013a, 2016). In this study, we report that a simple change in vector combination by using two EV plasmids to deliver M and K (M + K) instead of one (MK) leads to some 100-fold improvement in PB reprogramming. We further demonstrate that OCT4 and SOX2 linked by E2A (OS), but not other combinations such as OCT4-E2A-MYC (OM), OCT4-E2A-KLF4 (OK), and OCT4-E2A-SOX2-E2A-KLF4 (OSK), is the best choice for high-efficiency PB reprogramming.

RESULTS

Expression of MYC and KLF4 in Two Individual Episomal Vectors instead of One Dramatically Increases Reprogramming Efficiency

We have reported that the use of three EV plasmids to express Yamanaka factors and BCL-XL (OS + B + MK) leads to efficient generation of integration-free iPSCs from PB MNCs (Su et al., 2013a; Zhang, 2013). In our continuous efforts to optimize EV-mediated PB reprogramming, we cloned multiple vectors to express the five factors monocistronically or polycistronically (Figure 1A). In this study, frozen or freshly isolated PB MNCs were cultured in erythroid medium for 6 days to expand erythroid progenitors (Liu et al., 2014). After nucleofection with different combinations of EV plasmids, cells were cultured in hypoxia with Stemline-based serum-free erythroid medium, which was gradually changed to iPSC induction medium (Figure 1B). Between 6 and 14 days post-transfection, sodium butyrate, an inhibitor of histone deacetylases (HDACs) (Davie, 2003), was supplemented in the culture medium to enhance reprogramming (Figure 1B). We accidently found that using two vectors to express MYC and KLF4 separately leads to a striking increase in reprogramming efficiency (Figure 1C). Using four different PB samples (PM8, PM9, PM10, and PM11), we obtained 10–40 alkaline phosphatase (AP)-positive iPSC colonies from 1 × 10^6 cultured cells with the EV combination of OS + B + MK, whereas the OS + B + M + K combination gave rise to 1,000–3,000 iPSC colonies, representing an approximately 100-fold increase (Figure 1D). We also noticed that small iPSC colonies became visible in OS + B + M + K 1 week after transfection, and the average colony size was much larger on day 14 in this group than OS + B + MK (Figures 1C and S1A). These data suggest that the OS + B + M + K combination accelerates reprogramming dynamics and drastically enhances iPSC generation compared with the OS + B + MK combination.

Next, we asked if the increase in the number of iPSC colonies is at the expense of a decrease in stem cell quality. Staining the bulk population at day 14 with TRA-1-60, a pluripotency marker, showed no difference in the percentage of fully reprogrammed cells (~20% for both) (Figure 1E). Then, we picked ten colonies from each group and expanded iPSCs in E8 medium for five passages. The majority of colonies in both conditions can be expanded long term (data not shown). Fluorescence-activated cell sorting (FACS) analysis showed that >93% cells expressed TRA-1-60 or SSEA4, with no discernible differences

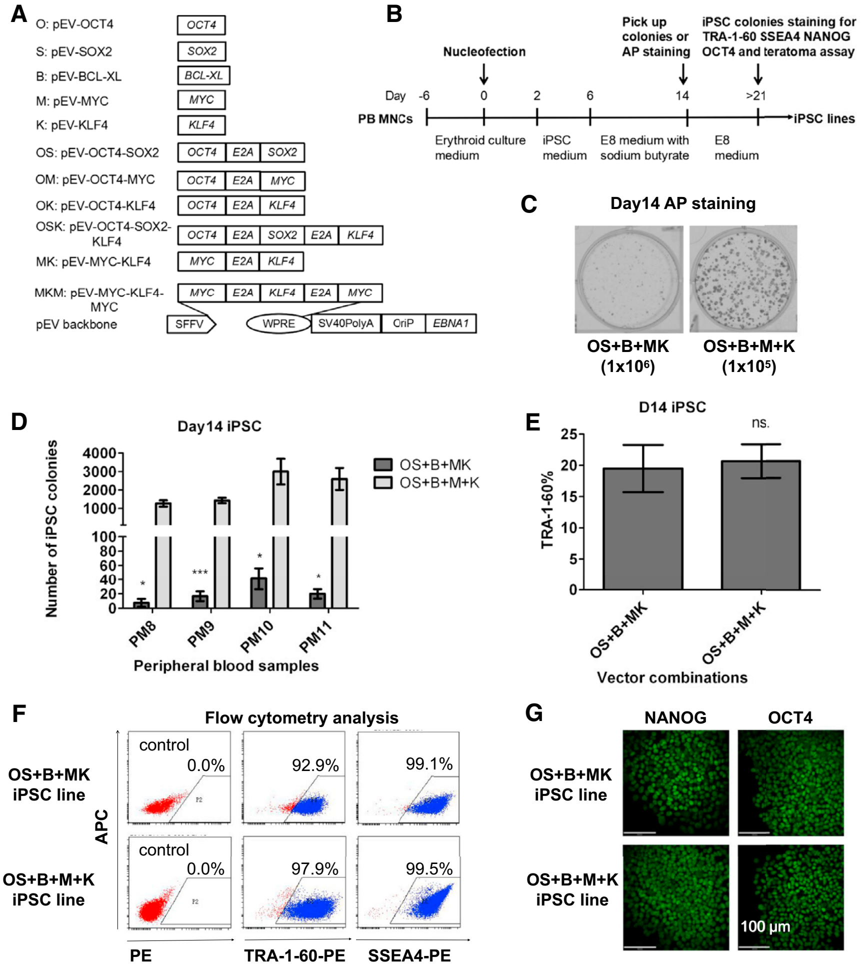

Figure 1. Expression of MYC and KLF4 in Two Individual Episomal Vectors instead of One Dramatically Increases the Reprogramming Efficiency of PB MNCs

(A) Schematic diagram of the EV plasmids. Reprogramming factors were cloned into the pEV backbone; their expression is driven by spleen focus-forming virus U3 promoter (SFFV). 2A (E2A) is a self-cleavage site derived from equine rhinitis A virus. Wpre, post-transcriptional regulatory element; SV40PolyA, polyadenylation signal from SV40 virus; OriP, EBV origin of replication; EBNA1, Epstein-Barr nuclear antigen 1, which plays essential roles in the replication and persistence of episomal plasmid in infected cells.

(B) Schematic illustration of the overall experimental design.

(C) Representative images of AP staining at 14 days after nucleofection of PB MNCs with EV plasmids. Note that the seeding number in each condition is different.

(D) Substantially increased reprogramming efficiency with the plasmid combination OS + B + M + K. Shown are numbers of iPSC colonies calculated from 1×10^6 PB MNCs (mean ± SEM, n = 3 independent experiments for each sample). $^*p < 0.05$; $^{***}p < 0.001$.

(E) Comparable levels of cells express TRA-1-60 between two groups. The whole populations of cells were analyzed by FACS at day 14 post-transfection (mean ± SEM, n = 5 independent experiments).

(F) Representative FACS diagrams of iPSCs expressing TRA-1-60 or SSEA4. iPSC colonies derived from indicated combinations of EV plasmids were analyzed at passage five.

(G) Representative confocal images of iPSC colonies expressing NANOG and OCT4. Scale bar, 100 μm.

See also Figure S1.

between groups OS + B + MK and OS + B + M + K (Figure 1F). Furthermore, iPSCs derived from the two combinations of EV plasmids showed expression of iPSC markers NANOG and OCT4 by confocal microscopy (Figures 1G, S1B, and S1C). Taken together, these data demonstrate that using two EV plasmids to express M and K (OS + B + M + K) instead of one vector (OS + B + MK) increases the reprogramming efficiency of PB MNCs by ~100-fold without affecting iPSC quality.

Dosage Optimization of Each Factor for High-Level Reprogramming

In our previous study, we showed that in the absence of M, OS + B + K also induces PB MNCs to pluripotency at a similar efficiency to that of OS + B + MK (Su et al., 2013a), but no systemic investigation on factor essentiality of the EV reprogramming system has been conducted. Here we attempted to address this question by omitting one factor each in the iPSC induction assays. We found that the absence of M, K, or B led to a substantial decrease in reprogramming efficiencies by a factor of ~100, ~10, and ~2–3, respectively (Figures 2A and S2A). Strikingly, depletion of either O or S from the combination induced a complete failure in reprogramming (Figures 2A and S2A). Together, these data demonstrate that all of the five factors are important for achieving high-level PB MNC reprogramming.

We further investigated the optimal dosage of each plasmid for PB reprogramming. In the above experiments, we used 2 μg of OS, 1 μg of B, 1 μg of M, and 1 μg of K. We first changed the dosage of OS, and found that halving or doubling the amount of OS plasmid did not obviously

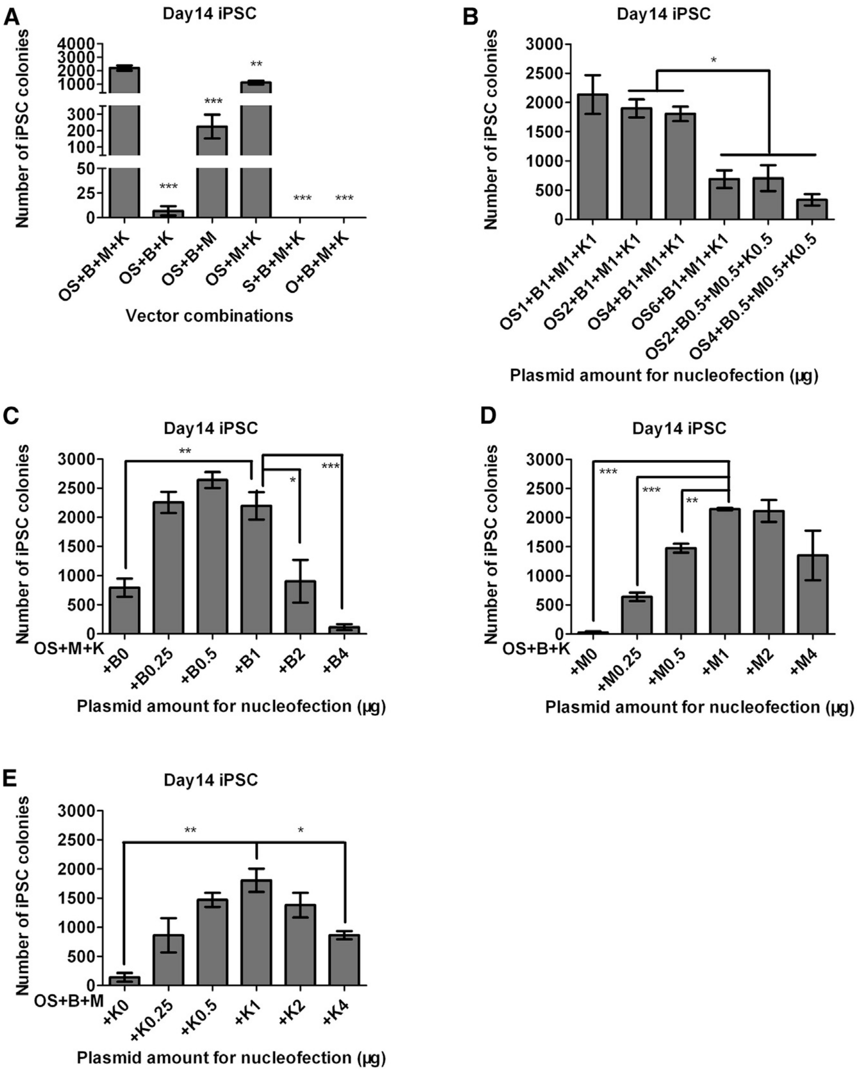

Figure 2. Optimal Amount of Each Factor Is Important for Achieving High-Level Reprogramming

(A) Each of the five factors is important for efficient reprogramming. Shown are numbers of iPSC colonies calculated from 1×10^6 PB MNCs (mean ± SEM, n = 3 independent experiments).

(B) Dosage effects of reprogramming factors. Shown are numbers of iPSC colonies from 1×10^6 PB MNCs transfected with the indicated amount of each plasmid (mean ± SEM, n = 3 independent experiments).

(C–E) Summary of dosage effects of BCL-XL (C), MYC (D), and KLF4 (E) on PB reprogramming. Shown are iPSC colony numbers from 1×10^6 PB MNCs (mean ± SEM, n = 3 independent experiments).

$^*p < 0.05$; $^{**}p < 0.01$; $^{***}p < 0.001$. See also Figure S2.

affect reprogramming efficiency, whereas a further increase from 4 μg to 6 μg significantly decreased reprogramming efficiency by ~60% (Figures 2B and S2B). This is likely because increased amount of plasmids during nucleofection induced more cell death. When we decreased the amount of B, M, and K each from 1 μg to 0.5 μg, we observed a 60%–80% decrease in reprogramming efficiency (Figures 2B and S2B). To further examine the dosage effects, we changed the amount of B, M, or K individually. We found that the optimal dosage for B is 0.25–0.5 μg, which increases the reprogramming efficiency by ~20% (Figures 2C and S2C). In comparison, the optimal dosages for both M and K are 1 μg, with either an increase or a decrease in the plasmid amount leading to a reduction in reprogramming efficiency (Figures 2D, 2E, S2D, and S2E). Taken together, to achieve high-level PB MNC reprogramming, the optimal vector dosage is 2 μg of OS + 0.5 μg of B + 1 μg of M + 1 μg of K.

Equimolar Expression of OCT4 and SOX2 and the Ratio of MYC and KLF4 Are the Most Critical Factors for Achieving High-Level Reprogramming

To systemically examine the effects of different vector combinations, we expressed the five genes individually or polycistronically. We observed a striking decrease in the number of iPSC colonies when OS was expressed by two vectors (O + S + B + M + K) instead of one (OS + B + M + K), although the percentages of TRA-1-60 positive cells in bulk populations showed no significant difference (Figures 3A and 3C). This finding was reproduced with four independent PB samples (PM8, PM9, PM10, and PM11); balanced expression of OS by linking them together with an E2A sequence led to a 20- to 40-fold increase in reprogramming efficiency compared with the use of two vectors to deliver O and S (O + S) (Figure 3B). We further examined the effects of conjugating OCT4 with other factors. Equimolar amounts of OCT4 and MYC (OM + B + S + K) led

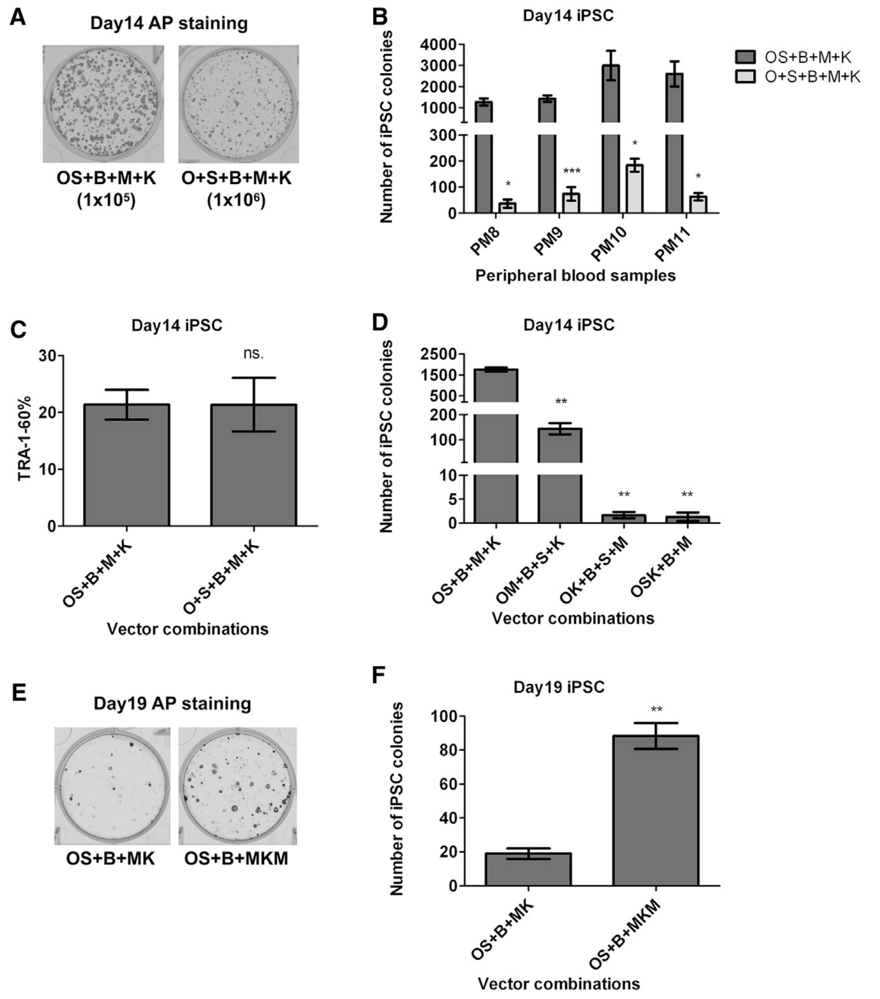

Figure 3. Equimolar Expression of OCT4 and SOX2 and Ratios of MYC and KLF4 Are the Most Critical Factors for Achieving High-Level Reprogramming

(A) Representative images of AP staining of iPSC induction with different combinations of plasmids. Note that the seeding cell numbers are different.

(B) Monocistronic expression of O and S induces a substantial reduction in reprogramming efficiency. Shown are numbers of iPSC colonies from 1×10^6 PB MNCs (mean ± SEM, n = 3 independent experiments for each sample).

(C) Comparable levels of cells express TRA-1-60 in two groups. The whole populations of cells were analyzed by FACS at day 14 post-transfection (mean ± SEM, n = 5 independent experiments).

(D) Polycistronic expression of O together with M or K leads to low-efficiency reprogramming. Shown are number of iPSC colonies from 1×10^6 PB MNCs (mean ± SEM, n = 3 independent experiments).

(E) Representative images of AP staining of iPSC from 1×10^6 PB MNCs. MK, pEV-SFFV-MYC-E2A-KLF4; MKM, pEV-SFFV-MYC-E2A-KLF4-E2A-MYC.

(F) Increased MYC:KLF4 ratio significantly increases reprogramming efficiency. Shown are AP positive colony numbers from 1×10^6 PB MNCs (mean ± SEM, n = 3 independent experiments).

*$p < 0.05$; **$p < 0.01$; ***$p < 0.001$. See also Figure S3.

to~90% reduction in reprogramming efficiency compared with OS + B + M + K, while bicistronic expression of O and K (OK + B + M + S) only generated a few iPSC colonies (Figures 3D and S3A). Similarly, balanced expression of OSK almost failed to reprogram PB MNCs to pluripotency (Figures 3D and S3A). Together, these data demonstrate that bicistronic expression of OCT4 and SOX2, but not other factor conjugations, is of critical importance for achieving high-level PB MNC reprogramming.

In contrast to balanced expression of O and S, equimolar expression of M and K (MK) suppresses PB MNC reprogramming (Figures 1C and 1D). To address the potential mechanism underlying the substantially decreased reprogramming capacity of MK, we analyzed the dynamic changes of *MYC* and *KLF4* mRNA expression levels after nucleofection of PB MNCs with OS + B + MK versus OS + B + M + K during the course of reprogramming. We observed a steady decrease of *MYC* expression in both groups but no obvious differences between the MK and the M + K group (Figures S3B and S3E). In contrast, we saw higher *KLF4* expression levels in the MK relative to the M + K group, and the differences increased to 3- to 10-fold between D5 and D13 post-transfection (Figures S3C and S3F). Plotting the ratio of *MYC* expression to *KLF4* expression showed that the M:K ratios were >100% higher in cells transfected with M + K versus MK, and the differences increased to 5- to 10-fold 1 week post-transfection (Figures S3D and S3G). These data strongly suggest that high-level *KLF4* expression and thereby lower M:K ratio may be responsible for the detrimental effects of the MK vector on reprogramming. To test this possibility, we cloned an MYC-E2A-KLF4-E2A-MYC (MKM) vector that encodes two copies of M and one copy of K, thus increasing the M:K ratio from 1:1 to 2:1. As expected, doubling of the M:K ratio led to a 4- to 5-fold increase in reprogramming efficiency (Figures 3E and 3F). However, reprogramming mediated by OS + B + MKM was still far less efficient than OS + B + M + K, likely because, at the later

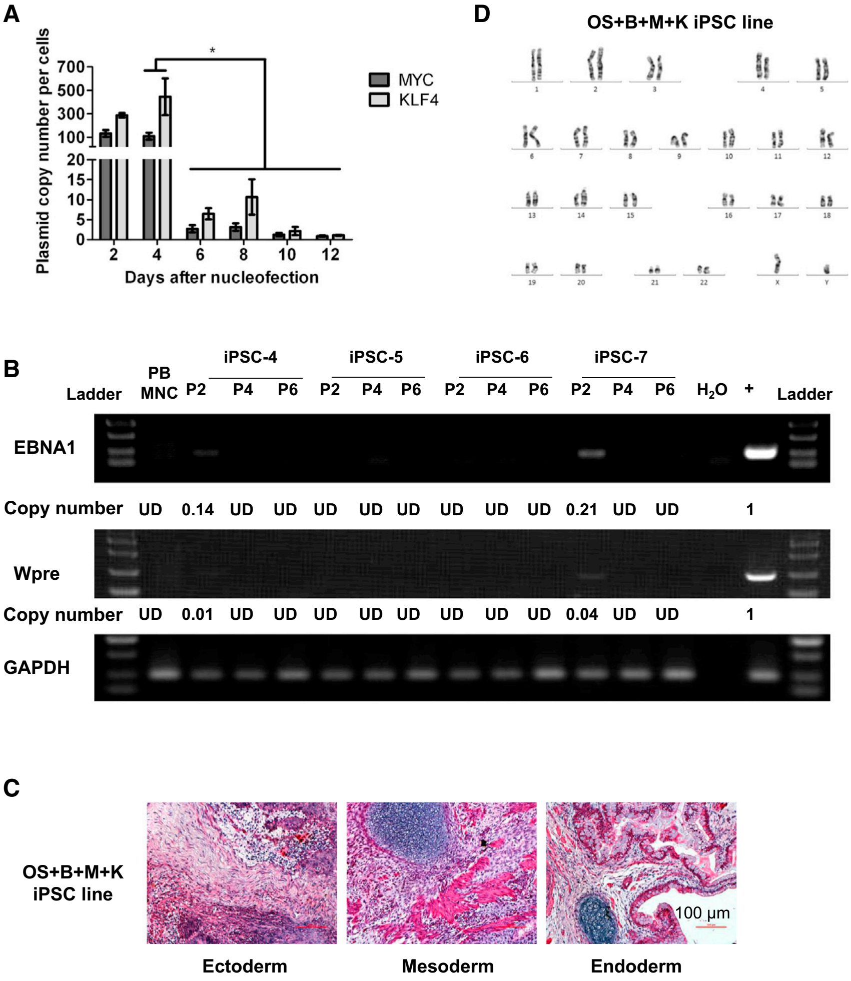

Figure 4. Characterization of iPSCs Generated with OS + B + M + K

(A) Dynamics of plasmid copy numbers after nucleofection. Data are normalized to a one-copy control (mean ± SEM, n = 4 independent experiments). *$p < 0.05$.

(B) Copy numbers of residual EV plasmids in iPSCs after two, four, or six passages. Specific primers for EBNA1 and Wpre were used to amplify episomal vectors. GAPDH was used as a DNA loading control. UD, undetectable. The positive lane indicates a one-copy control.

(C) H&E staining of teratomas comprising all three germ layers. Scale bar, 100 µm.

(D) G-band karyotyping shows a normal diploid 46, XY male karyotype.

stage of reprogramming, KLF4 expression in MKM-transfected cells was still too high to promote proliferation and progression of partially reprogrammed cells to full pluripotency. Taken together, expression of MYC and KLF4 with two individual vectors can maintain a dynamic ratio of both factors during the course of reprogramming, leading to elevated reprogramming efficiency.

Characterization of iPSCs Generated with the Improved Episomal Vector Combination OS + B + M + K

After transfection of EV, plasmids are gradually depleted from the cells, leading to the establishment of integration-free iPSCs weeks later, but the dynamics of EV depletion from reprogramming PB MNCs has not been reported. In this study, we were particularly interested in the depletion of MYC and KLF4 plasmids during the course of reprogramming. Cells were harvested from D2-12 at 2-day intervals for real-time PCR analysis using plasmid-specific primers. Genomic DNA from untransfected PB MNCs were used as a negative control, which showed no amplification of the PCR product. We found that there were more than 100 copies of M or K plasmids per cell in the first 4 days (Figure 4A). Of interest, this number precipitously dropped to less than ten 2 days later (Figure 4A), which coincided with a rapid cell proliferation. By day 10 post-transfection, there were less than two copies of M or K plasmids per cell in the bulk population (Figure 4A). We picked four colonies and tracked the changes in average copies of total plasmids in each cell. At passage two, only 0.1–0.2 copies per cells were detected in two out of four clones, while zero copies of plasmids were still present in four out of four clones at passages four and six (Figure 4B). These data suggest that EV plasmids are rapidly depleted from cells, and virtually no ectopic DNA can be detected at 1 month after transfection. Thus, integration-free iPSCs can be readily established with this approach.

Having examined the phenotype of generated PB iPSCs (Figure 1), we further characterized the established integration-free iPSC lines by teratoma assay and karyotyping. At 2 months after subcutaneous injection of iPSCs in

immunocompromised mice, we observed teratoma formation. Histological analysis of the teratomas showed the composition of tissues from all three primary germ layers (Figure 4C). To assess the genomic stability of the EV-generated integration-free iPSCs, an iPSC line was subjected to karyotype analysis after 20 passages of culture and found to display a normal karyotype (Figure 4D). These results demonstrate the non-integrating iPSC lines we generated are pluripotent and do not harbor obvious chromosomal abnormalities after long-term culture.

Finally, we compared the efficacy of our improved EV plasmids with another widely used integration-free reprogramming system, Sendai virus (SeV), which is commercially available at a high cost (>$2,000 per kit). Two weeks after transfection of the same amount of PB MNCs with EV or SeV, we observed more iPSC colonies in the EV condition relative to the SeV approach, but the difference did not reach statistical significance (Figures S1D and S1E). Of interest, EV-iPSC colonies were largely compact, whereas SeV-generated colonies appeared more diffused (Figure S1D). We also found that EV shows a higher success rate for establishing stable iPSC lines in our culture conditions: ~80% picked EV iPSCs can be passaged long term, whereas ~50% of SeV iPSCs spontaneously differentiated after 2–3 passages (not shown). Together, these results demonstrate that our improved combination of EV plasmids may provide a much better application prospect than SeV in the generation of integration-free iPSCs from adult PB.

DISCUSSION

Integration-free iPSCs hold great promise for clinical regenerative medicine. We have reported that an improved EV reprogramming system leads to a 10- to 100-fold increase in PB reprogramming compared with similar methods developed by other laboratories (Su et al., 2013a). However, the EV is still less efficient than another popular integration-free reprogramming vector system, the SeV. After a systemic investigation of vector combinations, we report in this study that a simple change using two individual vectors to express MYC and KLF4, leads to an additional ~100-fold increase in PB reprogramming than we previously reported (OS + MK + B) (Su et al., 2013a). The marked improvement can be ascribed to a relatively higher M:K ratio and lower KLF4 expression during the course of reprogramming. Another important factor for successful reprogramming is balanced expression of OCT4 and SOX2 mediated by a bicistronic vector. Other combinations such as OM + S + K + B, OK + S + M + B, or OSK + M + B show a significant decrease in reprogramming efficiency compared with OS + M + K + B, highlighting the importance of vector design. All five factors are critical, in particular O, S, M, and K. The iPSCs are indistinguishable from those generated with the previous protocol in expression of pluripotency markers and teratoma-forming ability. In addition, the iPSC lines show no residual plasmids and a normal karyotype after long-term culture.

Stoichiometry of reprogramming factors is one of the most critical factors for successful reprogramming. We report that equimolar expression of O and S leads to a 20- to 40-fold increase in reprogramming compared with monocistronic expression of O and S. This is likely due to inappropriate ratios in the vast majority of cells transfected with two vectors encoding O and S. Superficially speaking, balanced expression of mesoendoderm lineage specifier OCT4 and ectoderm lineage specifier SOX2 permits reprogramming to iPSCs, whereas unbalanced levels of O and S attenuate induced reprogramming to pluripotency (Montserrat et al., 2013; Shu et al., 2013). Mechanistically, O and S, along with NANOG, constitute the core transcriptional regulatory circuitry in iPSCs and embryonic stem cells (Boyer et al., 2005; Chen et al., 2008; Kim et al., 2008). After expression of O and S in transfected cells, they form a heterodimer (Remenyi et al., 2003) to bind the canonical motif, in which the SOX2 binding site is followed immediately by an octamer site (Ng et al., 2012), synergistically activating pluripotency factors like NANOG (Kuroda et al., 2005; Rodda et al., 2005). Multiple studies have also demonstrated that O in concert with S increases the transcriptional activity of OCT4 (Chew et al., 2005; Jang et al., 2012). In addition, more than 400 genes expressed in pluripotent stem cells are bound by both O and S to promote pluripotency and self-renewal (Boyer et al., 2005). All these data collectively provide an explanation for the remarkable increase in reprogramming efficiency mediated by equimolar expression of O and S.

MYC is not a pluripotency factor, but it substantially improves reprogramming dynamics, primarily because MYC accelerates cell proliferation (Sabo et al., 2014; Walz et al., 2014), providing an opportunity for the somatic genome to gradually reshape its epigenetic landscape. MYC is critical at an early stage in cell reprogramming by promoting the embryonic stem cell-like transcription pattern (Polo et al., 2012; Sridharan et al., 2009). Similarly to previous reports on fibroblast reprogramming (Nakagawa et al., 2008; Wernig et al., 2008), MYC increases PB reprogramming efficiency by ~100-fold. We observed partially reprogrammed cells in many combinations during the course of reprogramming. But with the addition of sodium butyrate, many partially reprogrammed cells were fully converted, leading to the expression of TRA-1-60 marker in ~20% cells 2 weeks after transfection in all the conditions we examined (Figures 1E and 3C). The use of MYC as a reprogramming factor was controversial. When a monocistronic

integrating vector was used to deliver MYC, reactivation of the silenced MYC vector in some cells induced tumors in iPSC-derived animals (Nakagawa et al., 2008; Okita et al., 2007; Wernig et al., 2008). However, when non-integrating plasmids were used, MYC was critical for the generation of high-quality iPSCs that have full developmental ability (Araki et al., 2011). In addition, we have reported that the inclusion of MYC in the factor combination does not significantly increase mutations during reprogramming (Su et al., 2013b). In this study, a high level of MYC expression is only observed in the first week of reprogramming after EV transfection (Figure S3), and no plasmid is detectable after four passages. Thus, we argue that the use of MYC is advantageous and does not engender obvious adverse effects in our system.

KLF4 is expressed at very low levels in mature human iPSCs, whereas low KLF4 expression only gives rise to partially reprogrammed iPSCs (Nishimura et al., 2014). Similarly, we observed that a decrease in KLF4 dosage significantly reduces the reprogramming efficiency, and its omission leads to an ~10-fold drop in PB reprogramming. KLF4 is proposed to be an upstream regulator of larger feedforward loops containing OCT4 and SOX2 (Kim et al., 2008), likely because KLF4 specifically recruits looping factor cohesin to the OCT4 distal enhancer, facilitating the formation of a higher-order chromatin structure for maintaining and inducing pluripotency (Wei et al., 2013).

Strikingly, we found that fixed stoichiometry of M and K disfavors PB reprogramming. When their stoichiometric relationships are fixed, the reprogramming efficiency is less impressive, although an increase of M:K from 1:1 to 2:1 leads to a 4-fold increase in reprogramming (Figure 3F). We also found that high-efficiency reprogramming by OS + B + M + K is associated with significantly decreased *KLF4* mRNA levels (and high M:K ratios) compared with the low-efficiency combination OS + B + MK (Figure S3). These data strongly suggest a differential requirement of KLF4 during the early and late stages of reprogramming—high-level expression at the beginning to facilitate cell transformation, followed by low-level expression to allow for rapid cell proliferation. It is reported that overexpression of KLF4 results in the inhibition of DNA synthesis (McConnell et al., 2007) and activation of anti-proliferative genes (Rowland and Peeper, 2006), and we also found that transduction of hematopoietic cells with KLF4 restrains cell proliferation (not shown). We propose that when M and K are expressed with two vectors, the optimal M:K ratio is achieved by self-adaptation of transfected cells—cells with the right amount of KLF4 and the right ratio of M:K at the right time are preferentially reprogrammed and selected for due to proliferative advantage of these cells. As a result, expression of M and K with two plasmids instead of one remarkably enhanced reprogramming.

Many non-integrating reprogramming strategies have been investigated over the past 8 years, such as minicircle plasmids, synthetic mRNA/miRNA, proteins, and small molecules. However, all these approaches are labor intensive, time consuming, and often inefficient. Recently, much attention has been focused on two simple vector systems: SeV and EV. After only one infection or transfection, dozens or even hundreds of iPSC colonies can be attained 2–3 weeks later. Currently, SeV is 10- to 100-times more efficient (Schlaeger et al., 2015), whereas EV is more affordable and does not demand onerous administrative approval. The cost of EV plasmid preparation and nucleofection reagent is ~$10 per experiment, which is ~90% lower than purchasing the SeV reprogramming kit. Using our improved EV plasmid combination, which may outcompete SeV in reprogramming efficiency, the primary advantage of SeV vanishes. A recent comparison of EV versus SeV shows that EV iPSCs have a higher occurrence of aneuploidies (12% versus 5%) (Schlaeger et al., 2015). However, an alternative explanation of the data is that the increased abnormalities may have nothing to do with the EV itself, but rather the use of shP53 in the Yamanaka EV system. It is most likely that suppression of p53, a guardian of ploidy (Aylon and Oren, 2011), accounts for the increased occurrence of aneuploidies. With this in mind, we did not include shP53 or SV40 big T protein in our system. Instead, we used an anti-apoptotic factor, BCL-XL, which has no reported link with genetic abnormalities. Accordingly, human iPSC lines generated with Yamanaka factors together with BCL-XL display a normal karyotype.

Taken together, the combination of EVs (OS + M + K + B) meets the dynamic stoichiometry requirements for reprogramming factors, leading to a remarkable enhancement in the transition of PB MNCs to pluripotency. The improved EV system is comparable with SeV in reprogramming efficiency, making the affordable EV approach more attractive and thus eradicating the last barrier to the broad application of EV plasmids in translational cellular reprogramming.

EXPERIMENTAL PROCEDURES

PB and MNC Isolation

Human PB was obtained from anonymous adult donors with no identification information available from Tianjin Blood Center with approval of the local research ethics committee. MNCs were obtained by standard density gradient centrifugation with Ficoll-Hypaque (1.077 g/ml) (G&E Healthcare; cat. no. 17-1440-03) at room temperature as previously described (Zhang, 2013).

Episomal Vectors

Inserts of OCT4-E2A-SOX2 (OS), KLF4 (K), BCL-XL (B), MYC-E2A-KLF4 (MK), OCT4-E2A-MYC (OM), OCT4-E2A-KLF4 (OK), and

OCT4-E2A-SOX2-E2A-KLF4 (OSK) were cloned into an EV plasmid backbone bearing the SFFV promoter, Wpre, PolyA, oriP, and EBNA1 elements as described previously (Su et al., 2013a; Zhang, 2013). To drive the expression of two genes, a self-cleaving peptide sequence from equine rhinitis A virus (E2A) was used to link the two genes. The sequence of E2A we used is CAG TGT ACT AAT TAT GCT CTC TTG AAA TTG GCT GGA GAT GTT GAG AGC AAC CCA GGT CCC. EV plasmids OCT4 (O), SOX2 (S), and MYC (M) were constructed by inserting the open reading frames of OCT4, SOX2, and MYC into the EV backbone, respectively. MYC-E2A-KLF4-E2A-MYC (MKM) was constructed by assembling MK with the E2A-MYC inert. All the inserts of the cloned vectors were verified by sequencing.

Reprogramming of PB MNCs to Pluripotency

PB MNCs were cultured in erythroid medium composed of Stemline II Hematopoietic Stem Cell Expansion Medium (Sigma; S0192) supplemented with 100 ng/ml stem cell factor (Peprotech; 300-07), 10 ng/ml interleukin-3 (Peprotech; AF-200-03), 2 U/ml eryrthropoietin (Peprotech; 100-64), 20 ng/ml insulin growth factor-1 (Peprotech; 100-11), 1 μM dexamethasone (Sigma; D4902) and 0.2 mM 1-thioglycerol (Sigma; M6145). After 6 days of culture, 2×10^6 cells were nucleofected with indicated plasmids and 5×10^4 to 1×10^6 of cells were plated in gelatin-treated 6-well plates with mitomycin-inactivated murine embryonic fibroblast feeder cells seeded 1 day before nucleofection. At days 0–2 after nucleofection, PB MNCs were cultured in erythroid medium. On day 2, we added to each well 2 ml of iPSC induction medium, composed of Knockout DMEM/F12 (Gibco; 112660-012) with 1× L-glutamine (Gibco; 25030-081), 1× penicillin/streptomycin (Gibco; 15140-122), 1× non-essential amino acids solution (Gibco; 11140-050), 50 ng/ml fibroblast growth factor 2 (Peprotech; 100-18B), 1× ITS (Gibco; 41400-045), and 50 μg/ml ascorbic acid (Sigma; 49752). At day 4, the culture was refreshed with 2 ml of iPSC induction medium. Starting on day 6, cells were fed with 2 ml of fresh E8 medium (Gibco; A1517001) supplemented with 0.25 mM sodium butyrate every 2 days until day 14. For long-term culture, iPSCs were maintained in Matrigel-precoated-well plates and refreshed with E8 medium daily.

Determination of Dynamic Changes of Plasmid Copy Numbers

PB MNCs after nucleofection were cultured for indicated days and genomic DNA was extracted. Plasmid copy numbers of MYC and KLF4 were analyzed by real-time PCR using MYC or KLF4 plasmid-specific primers (Table S1). Genomic DNA from untransfected PB MNCs was used as a negative control. To quantify the EV copy number, 1.6 pg of M or K plasmid was mixed with 1 μg of gDNA from untransfected PB MNCs to mimic cells with one copy of EV plasmid per cell.

Generation of iPSCs by Sendai Viral Vector

Sendai viral reprogramming was performed using a CytoTune-iPS Reprogramming Kit (Invitrogen; A13780) according to the manufacturer's protocol. After 6 days of culture in erythroid medium, as detailed above for EV-induced reprogramming, 2×10^5 PB MNCs were infected with 25 μl each of Sendai viral vector expressing one of the four Yamanaka factors (OCT4, SOX2, KLF4, and MYC). On the next day, PB MNCs were washed with PBS and half the amount of cells was plated into 6-well plates with mitomycin-inactivated murine embryonic fibroblast feeder cells seeded 1 day before. Starting on day 2, PB MNCs were cultured in the same conditions as EV-induced reprogramming.

Statistical Analysis

Data are presented as means and SEM. Two-tailed Student's t test was performed. p Values of < 0.05 were considered statistically significant. *$p < 0.05$; **$p < 0.01$; ***$p < 0.001$; ns, no significance.

Details of the experimental methods are included in the Supplemental Experimental Procedures.

SUPPLEMENTAL INFORMATION

Supplemental Information includes Supplemental Experimental Procedures, three figures, and one table and can be found with this article online at http://dx.doi.org/10.1016/j.stemcr.2016.04.005.

AUTHOR CONTRIBUTIONS

X.B.Z. conceived the project. W.W., J.P.Z., J.X., R.J.S., A.N., G.Z.J., and X.B.Z. conducted the experiments. W.W., J.P.Z., J.X., W.Y., T.C., and X.B.Z. analyzed the results. W.W., T.C., and X.B.Z. wrote the paper. All authors reviewed the manuscript.

ACKNOWLEDGMENTS

This work was supported by the Ministry of Science and Technology of China (2015CB964902, 2013CB966902 and 2012CB966601), the National Natural Science Foundation of China (81500148, 81570164 and 81421002), and the Loma Linda University School of Medicine GCAT grant (2015).

Received: February 19, 2016
Revised: April 5, 2016
Accepted: April 6, 2016
Published: May 5, 2016

REFERENCES

Aasen, T., Raya, A., Barrero, M.J., Garreta, E., Consiglio, A., Gonzalez, F., Vassena, R., Bilic, J., Pekarik, V., Tiscornia, G., et al. (2008). Efficient and rapid generation of induced pluripotent stem cells from human keratinocytes. Nat. Biotechnol. *26*, 1276–1284.

Abyzov, A., Mariani, J., Palejev, D., Zhang, Y., Haney, M.S., Tomasini, L., Ferrandino, A.F., Rosenberg Belmaker, L.A., Szekely, A., Wilson, M., et al. (2012). Somatic copy number mosaicism in human skin revealed by induced pluripotent stem cells. Nature *492*, 438–442.

Agu, C.A., Soares, F.A., Alderton, A., Patel, M., Ansari, R., Patel, S., Forrest, S., Yang, F., Lineham, J., Vallier, L., et al. (2015). Successful generation of human induced pluripotent stem cell lines from blood samples held at room temperature for up to 48 hr. Stem Cell Rep. *5*, 660–671.

Araki, R., Hoki, Y., Uda, M., Nakamura, M., Jincho, Y., Tamura, C., Sunayama, M., Ando, S., Sugiura, M., Yoshida, M.A., et al. (2011). Crucial role of c-Myc in the generation of induced pluripotent stem cells. Stem Cells *29*, 1362–1370.

Aylon, Y., and Oren, M. (2011). p53: guardian of ploidy. Mol. Oncol. *5*, 315–323.

Boyer, L.A., Lee, T.I., Cole, M.F., Johnstone, S.E., Levine, S.S., Zucker, J.P., Guenther, M.G., Kumar, R.M., Murray, H.L., Jenner, R.G., et al. (2005). Core transcriptional regulatory circuitry in human embryonic stem cells. Cell *122*, 947–956.

Broxmeyer, H.E., Lee, M.R., Hangoc, G., Cooper, S., Prasain, N., Kim, Y.J., Mallett, C., Ye, Z., Witting, S., Cornetta, K., et al. (2011). Hematopoietic stem/progenitor cells, generation of induced pluripotent stem cells, and isolation of endothelial progenitors from 21- to 23.5-year cryopreserved cord blood. Blood *117*, 4773–4777.

Carey, B.W., Markoulaki, S., Hanna, J.H., Faddah, D.A., Buganim, Y., Kim, J., Ganz, K., Steine, E.J., Cassady, J.P., Creyghton, M.P., et al. (2011). Reprogramming factor stoichiometry influences the epigenetic state and biological properties of induced pluripotent stem cells. Cell Stem Cell *9*, 588–598.

Chen, X., Xu, H., Yuan, P., Fang, F., Huss, M., Vega, V.B., Wong, E., Orlov, Y.L., Zhang, W., Jiang, J., et al. (2008). Integration of external signaling pathways with the core transcriptional network in embryonic stem cells. Cell *133*, 1106–1117.

Chew, J.L., Loh, Y.H., Zhang, W., Chen, X., Tam, W.L., Yeap, L.S., Li, P., Ang, Y.S., Lim, B., Robson, P., et al. (2005). Reciprocal transcriptional regulation of Pou5f1 and Sox2 via the Oct4/Sox2 complex in embryonic stem cells. Mol. Cell Biol. *25*, 6031–6046.

Chou, B.K., Mali, P., Huang, X., Ye, Z., Dowey, S.N., Resar, L.M., Zou, C., Zhang, Y.A., Tong, J., and Cheng, L. (2011). Efficient human iPS cell derivation by a non-integrating plasmid from blood cells with unique epigenetic and gene expression signatures. Cell Res. *21*, 518–529.

Chou, B.K., Gu, H., Gao, Y., Dowey, S.N., Wang, Y., Shi, J., Li, Y., Ye, Z., Cheng, T., and Cheng, L. (2015). A facile method to establish human induced pluripotent stem cells from adult blood cells under feeder-free and xeno-free culture conditions: a clinically compliant approach. Stem Cells Transl. Med. *4*, 320–332.

Davie, J.R. (2003). Inhibition of histone deacetylase activity by butyrate. J. Nutr. *133*, 2485S–2493S.

de Felipe, P., Luke, G.A., Hughes, L.E., Gani, D., Halpin, C., and Ryan, M.D. (2006). E unum pluribus: multiple proteins from a self-processing polyprotein. Trends Biotechnol. *24*, 68–75.

Diecke, S., Lu, J., Lee, J., Termglinchan, V., Kooreman, N.G., Burridge, P.W., Ebert, A.D., Churko, J.M., Sharma, A., Kay, M.A., et al. (2015). Novel codon-optimized mini-intronic plasmid for efficient, inexpensive, and xeno-free induction of pluripotency. Sci. Rep. *5*, 8081.

Dorigo, O., Gil, J.S., Gallaher, S.D., Tan, B.T., Castro, M.G., Lowenstein, P.R., Calos, M.P., and Berk, A.J. (2004). Development of a novel helper-dependent adenovirus-Epstein-Barr virus hybrid system for the stable transformation of mammalian cells. J. Virol. *78*, 6556–6566.

Dowey, S.N., Huang, X., Chou, B.K., Ye, Z., and Cheng, L. (2012). Generation of integration-free human induced pluripotent stem cells from postnatal blood mononuclear cells by plasmid vector expression. Nat. Protoc. *7*, 2013–2021.

Hu, K., Yu, J., Suknuntha, K., Tian, S., Montgomery, K., Choi, K.D., Stewart, R., Thomson, J.A., and Slukvin, I.I. (2011). Efficient generation of transgene-free induced pluripotent stem cells from normal and neoplastic bone marrow and cord blood mononuclear cells. Blood *117*, e109–e119.

Jang, H., Kim, T.W., Yoon, S., Choi, S.Y., Kang, T.W., Kim, S.Y., Kwon, Y.W., Cho, E.J., and Youn, H.D. (2012). O-GlcNAc regulates pluripotency and reprogramming by directly acting on core components of the pluripotency network. Cell Stem Cell *11*, 62–74.

Kim, J., Chu, J., Shen, X., Wang, J., and Orkin, S.H. (2008). An extended transcriptional network for pluripotency of embryonic stem cells. Cell *132*, 1049–1061.

Kim, S.I., Oceguera-Yanez, F., Hirohata, R., Linker, S., Okita, K., Yamada, Y., Yamamoto, T., Yamanaka, S., and Woltjen, K. (2015). KLF4 N-terminal variance modulates induced reprogramming to pluripotency. Stem Cell Rep. *4*, 727–743.

Kuroda, T., Tada, M., Kubota, H., Kimura, H., Hatano, S.Y., Suemori, H., Nakatsuji, N., and Tada, T. (2005). Octamer and Sox elements are required for transcriptional cis regulation of Nanog gene expression. Mol. Cell Biol. *25*, 2475–2485.

Liu, S.P., Li, Y.X., Xu, J., Gu, H.H., Zhang, H.Y., Liang, H.Y., Liu, H.Z., Zhang, X.B., Cheng, T., and Yuan, W.P. (2014). An improved method for generating integration-free human induced pluripotent stem cells. Zhongguo Shi Yan Xue Ye Xue Za Zhi *22*, 580–587, [in Chinese].

Lo, C.A., Kays, I., Emran, F., Lin, T.J., Cvetkovska, V., and Chen, B.E. (2015). Quantification of protein levels in single living cells. Cell Rep. *13*, 2634–2644.

Loh, Y.H., Agarwal, S., Park, I.H., Urbach, A., Huo, H., Heffner, G.C., Kim, K., Miller, J.D., Ng, K., and Daley, G.Q. (2009). Generation of induced pluripotent stem cells from human blood. Blood *113*, 5476–5479.

Loh, Y.H., Hartung, O., Li, H., Guo, C., Sahalie, J.M., Manos, P.D., Urbach, A., Heffner, G.C., Grskovic, M., Vigneault, F., et al. (2010). Reprogramming of T cells from human peripheral blood. Cell Stem Cell *7*, 15–19.

Mack, A.A., Kroboth, S., Rajesh, D., and Wang, W.B. (2011). Generation of induced pluripotent stem cells from CD34+ cells across blood drawn from multiple donors with non-integrating episomal vectors. PLoS One *6*, e27956.

Mali, P., Ye, Z., Hommond, H.H., Yu, X., Lin, J., Chen, G., Zou, J., and Cheng, L. (2008). Improved efficiency and pace of generating induced pluripotent stem cells from human adult and fetal fibroblasts. Stem Cells *26*, 1998–2005.

Mali, P., Chou, B.K., Yen, J., Ye, Z., Zou, J., Dowey, S., Brodsky, R.A., Ohm, J.E., Yu, W., Baylin, S.B., et al. (2010). Butyrate greatly enhances derivation of human induced pluripotent stem cells by promoting epigenetic remodeling and the expression of pluripotency-associated genes. Stem Cells *28*, 713–720.

McConnell, B.B., Ghaleb, A.M., Nandan, M.O., and Yang, V.W. (2007). The diverse functions of Kruppel-like factors 4 and 5 in epithelial biology and pathobiology. Bioessays *29*, 549–557.

Meng, X., Neises, A., Su, R.J., Payne, K.J., Ritter, L., Gridley, D.S., Wang, J., Sheng, M., Lau, K.H., Baylink, D.J., et al. (2012). Efficient reprogramming of human cord blood CD34+ cells into induced pluripotent stem cells with OCT4 and SOX2 alone. Mol. Ther. *20*, 408–416.

Meraviglia, V., Zanon, A., Lavdas, A.A., Schwienbacher, C., Silipigni, R., Di Segni, M., Chen, H.S., Pramstaller, P.P., Hicks, A.A., and Rossini, A. (2015). Generation of induced pluripotent stem cells from frozen buffy coats using non-integrating episomal plasmids. J. Vis. Exp., e52885.

Merling, R.K., Sweeney, C.L., Choi, U., De Ravin, S.S., Myers, T.G., Otaizo-Carrasquero, F., Pan, J., Linton, G., Chen, L., Koontz, S., et al. (2013). Transgene-free iPSCs generated from small volume peripheral blood nonmobilized CD34+ cells. Blood *121*, e98–e107.

Montserrat, N., Nivet, E., Sancho-Martinez, I., Hishida, T., Kumar, S., Miquel, L., Cortina, C., Hishida, Y., Xia, Y., Esteban, C.R., et al. (2013). Reprogramming of human fibroblasts to pluripotency with lineage specifiers. Cell Stem Cell *13*, 341–350.

Nakagawa, M., Koyanagi, M., Tanabe, K., Takahashi, K., Ichisaka, T., Aoi, T., Okita, K., Mochiduki, Y., Takizawa, N., and Yamanaka, S. (2008). Generation of induced pluripotent stem cells without Myc from mouse and human fibroblasts. Nat. Biotechnol. *26*, 101–106.

Ng, C.K., Li, N.X., Chee, S., Prabhakar, S., Kolatkar, P.R., and Jauch, R. (2012). Deciphering the Sox-Oct partner code by quantitative cooperativity measurements. Nucleic Acids Res. *40*, 4933–4941.

Nishimura, T., Kaneko, S., Kawana-Tachikawa, A., Tajima, Y., Goto, H., Zhu, D., Nakayama-Hosoya, K., Iriguchi, S., Uemura, Y., Shimizu, T., et al. (2013). Generation of rejuvenated antigen-specific T cells by reprogramming to pluripotency and redifferentiation. Cell Stem Cell *12*, 114–126.

Nishimura, K., Kato, T., Chen, C., Oinam, L., Shiomitsu, E., Ayakawa, D., Ohtaka, M., Fukuda, A., Nakanishi, M., and Hisatake, K. (2014). Manipulation of KLF4 expression generates iPSCs paused at successive stages of reprogramming. Stem Cell Rep. *3*, 915–929.

Okita, K., Ichisaka, T., and Yamanaka, S. (2007). Generation of germline-competent induced pluripotent stem cells. Nature *448*, 313–317.

Okita, K., Matsumura, Y., Sato, Y., Okada, A., Morizane, A., Okamoto, S., Hong, H., Nakagawa, M., Tanabe, K., Tezuka, K., et al. (2011). A more efficient method to generate integration-free human iPS cells. Nat. Methods *8*, 409–412.

Okita, K., Yamakawa, T., Matsumura, Y., Sato, Y., Amano, N., Watanabe, A., Goshima, N., and Yamanaka, S. (2013). An efficient nonviral method to generate integration-free human-induced pluripotent stem cells from cord blood and peripheral blood cells. Stem Cells *31*, 458–466.

Papapetrou, E.P., Tomishima, M.J., Chambers, S.M., Mica, Y., Reed, E., Menon, J., Tabar, V., Mo, Q., Studer, L., and Sadelain, M. (2009). Stoichiometric and temporal requirements of Oct4, Sox2, Klf4, and c-Myc expression for efficient human iPSC induction and differentiation. Proc. Natl. Acad. Sci. USA *106*, 12759–12764.

Park, I.H., Zhao, R., West, J.A., Yabuuchi, A., Huo, H., Ince, T.A., Lerou, P.H., Lensch, M.W., and Daley, G.Q. (2008). Reprogramming of human somatic cells to pluripotency with defined factors. Nature *451*, 141–146.

Polo, J.M., Anderssen, E., Walsh, R.M., Schwarz, B.A., Nefzger, C.M., Lim, S.M., Borkent, M., Apostolou, E., Alaei, S., Cloutier, J., et al. (2012). A molecular roadmap of reprogramming somatic cells into iPS cells. Cell *151*, 1617–1632.

Remenyi, A., Lins, K., Nissen, L.J., Reinbold, R., Scholer, H.R., and Wilmanns, M. (2003). Crystal structure of a POU/HMG/DNA ternary complex suggests differential assembly of Oct4 and Sox2 on two enhancers. Genes Dev. *17*, 2048–2059.

Rodda, D.J., Chew, J.L., Lim, L.H., Loh, Y.H., Wang, B., Ng, H.H., and Robson, P. (2005). Transcriptional regulation of nanog by OCT4 and SOX2. J. Biol. Chem. *280*, 24731–24737.

Rowland, B.D., and Peeper, D.S. (2006). KLF4, p21 and context-dependent opposing forces in cancer. Nat. Rev. Cancer *6*, 11–23.

Sabo, A., Kress, T.R., Pelizzola, M., de Pretis, S., Gorski, M.M., Tesi, A., Morelli, M.J., Bora, P., Doni, M., Verrecchia, A., et al. (2014). Selective transcriptional regulation by Myc in cellular growth control and lymphomagenesis. Nature *511*, 488–492.

Schlaeger, T.M., Daheron, L., Brickler, T.R., Entwisle, S., Chan, K., Cianci, A., DeVine, A., Ettenger, A., Fitzgerald, K., Godfrey, M., et al. (2015). A comparison of non-integrating reprogramming methods. Nat. Biotechnol. *33*, 58–63.

Shu, J., Wu, C., Wu, Y., Li, Z., Shao, S., Zhao, W., Tang, X., Yang, H., Shen, L., Zuo, X., et al. (2013). Induction of pluripotency in mouse somatic cells with lineage specifiers. Cell *153*, 963–975.

Sridharan, R., Tchieu, J., Mason, M.J., Yachechko, R., Kuoy, E., Horvath, S., Zhou, Q., and Plath, K. (2009). Role of the murine reprogramming factors in the induction of pluripotency. Cell *136*, 364–377.

Staerk, J., Dawlaty, M.M., Gao, Q., Maetzel, D., Hanna, J., Sommer, C.A., Mostoslavsky, G., and Jaenisch, R. (2010). Reprogramming of human peripheral blood cells to induced pluripotent stem cells. Cell Stem Cell *7*, 20–24.

Su, R.J., Baylink, D.J., Neises, A., Kiroyan, J.B., Meng, X., Payne, K.J., Tschudy-Seney, B., Duan, Y., Appleby, N., Kearns-Jonker, M., et al. (2013a). Efficient generation of integration-free iPS cells from human adult peripheral blood using BCL-XL together with Yamanaka factors. PLoS One *8*, e64496.

Su, R.J., Yang, Y., Neises, A., Payne, K.J., Wang, J., Viswanathan, K., Wakeland, E.K., Fang, X., and Zhang, X.B. (2013b). Few single nucleotide variations in exomes of human cord blood induced pluripotent stem cells. PLoS One *8*, e59908.

Su, R.J., Neises, A., and Zhang, X.B. (2016). Generation of iPS cells from human peripheral blood mononuclear cells using episomal vectors. Methods Mol. Biol. *1357*, 57–69.

Takahashi, K., Tanabe, K., Ohnuki, M., Narita, M., Ichisaka, T., Tomoda, K., and Yamanaka, S. (2007). Induction of pluripotent stem cells from adult human fibroblasts by defined factors. Cell *131*, 861–872.

Takahashi, K., and Yamanaka, S. (2006). Induction of pluripotent stem cells from mouse embryonic and adult fibroblast cultures by defined factors. Cell *126*, 663–676.

Wakao, H., Yoshikiyo, K., Koshimizu, U., Furukawa, T., Enomoto, K., Matsunaga, T., Tanaka, T., Yasutomi, Y., Yamada, T., Minakami, H., et al. (2013). Expansion of functional human mucosal-associated invariant T cells via reprogramming to pluripotency and redifferentiation. Cell Stem Cell *12*, 546–558.

Walz, S., Lorenzin, F., Morton, J., Wiese, K.E., von Eyss, B., Herold, S., Rycak, L., Dumay-Odelot, H., Karim, S., Bartkuhn, M., et al. (2014). Activation and repression by oncogenic MYC shape tumour-specific gene expression profiles. Nature *511*, 483–487.

Wei, Z., Gao, F., Kim, S., Yang, H., Lyu, J., An, W., Wang, K., and Lu, W. (2013). Klf4 organizes long-range chromosomal interactions with the oct4 locus in reprogramming and pluripotency. Cell Stem Cell *13*, 36–47.

Wernig, M., Meissner, A., Cassady, J.P., and Jaenisch, R. (2008). c-Myc is dispensable for direct reprogramming of mouse fibroblasts. Cell Stem Cell *2*, 10–12.

Yang, S., Cohen, C.J., Peng, P.D., Zhao, Y., Cassard, L., Yu, Z., Zheng, Z., Jones, S., Restifo, N.P., Rosenberg, S.A., et al. (2008). Development of optimal bicistronic lentiviral vectors facilitates high-level TCR gene expression and robust tumor cell recognition. Gene Ther. *15*, 1411–1423.

Yu, J., Vodyanik, M.A., Smuga-Otto, K., Antosiewicz-Bourget, J., Frane, J.L., Tian, S., Nie, J., Jonsdottir, G.A., Ruotti, V., Stewart, R., et al. (2007). Induced pluripotent stem cell lines derived from human somatic cells. Science *318*, 1917–1920.

Yu, J., Hu, K., Smuga-Otto, K., Tian, S., Stewart, R., Slukvin, I.I., and Thomson, J.A. (2009). Human induced pluripotent stem cells free of vector and transgene sequences. Science *324*, 797–801.

Yu, J., Chau, K.F., Vodyanik, M.A., Jiang, J., and Jiang, Y. (2011). Efficient feeder-free episomal reprogramming with small molecules. PLoS One *6*, e17557.

Zhang, X.B. (2013). Cellular reprogramming of human peripheral blood cells. Genomics Proteomics Bioinformatics *11*, 264–274.

Stem Cell Reports, Volume *6*

Supplemental Information

Enhanced Generation of Integration-free iPSCs from Human Adult Peripheral Blood Mononuclear Cells with an Optimal Combination of Episomal Vectors

Wei Wen, Jian-Ping Zhang, Jing Xu, Ruijun Jeanna Su, Amanda Neises, Guang-Zhen Ji, Weiping Yuan, Tao Cheng, and Xiao-Bing Zhang

Stem Cell Reports, Volume 6

Supplemental Information

Enhanced Generation of Integration-free iPS Cells from Human Adult Peripheral Blood Mononuclear Cells with an Optimal Combination of Episomal Vectors

Wei Wen[1*], Jian-Ping Zhang[1*], Jing Xu[1*], Ruijun Jeanna Su[2], Amanda Neises[2], Guang-Zhen Ji[1], Weiping Yuan[1], Tao Cheng[1], Xiao-Bing Zhang[1,2]

[1] State Key Laboratory of Experimental Hematology, Institute of Hematology and Blood Disease Hospital, Chinese Academy of Medical Sciences and Peking Union Medical College, Tianjin 300020,China

[2] Department of Medicine, Loma Linda University, Loma Linda, CA 92350, USA

*These authors contributed equally to this work.

SUPPLEMENTAL FIGURES AND FIGURE LEGENDS

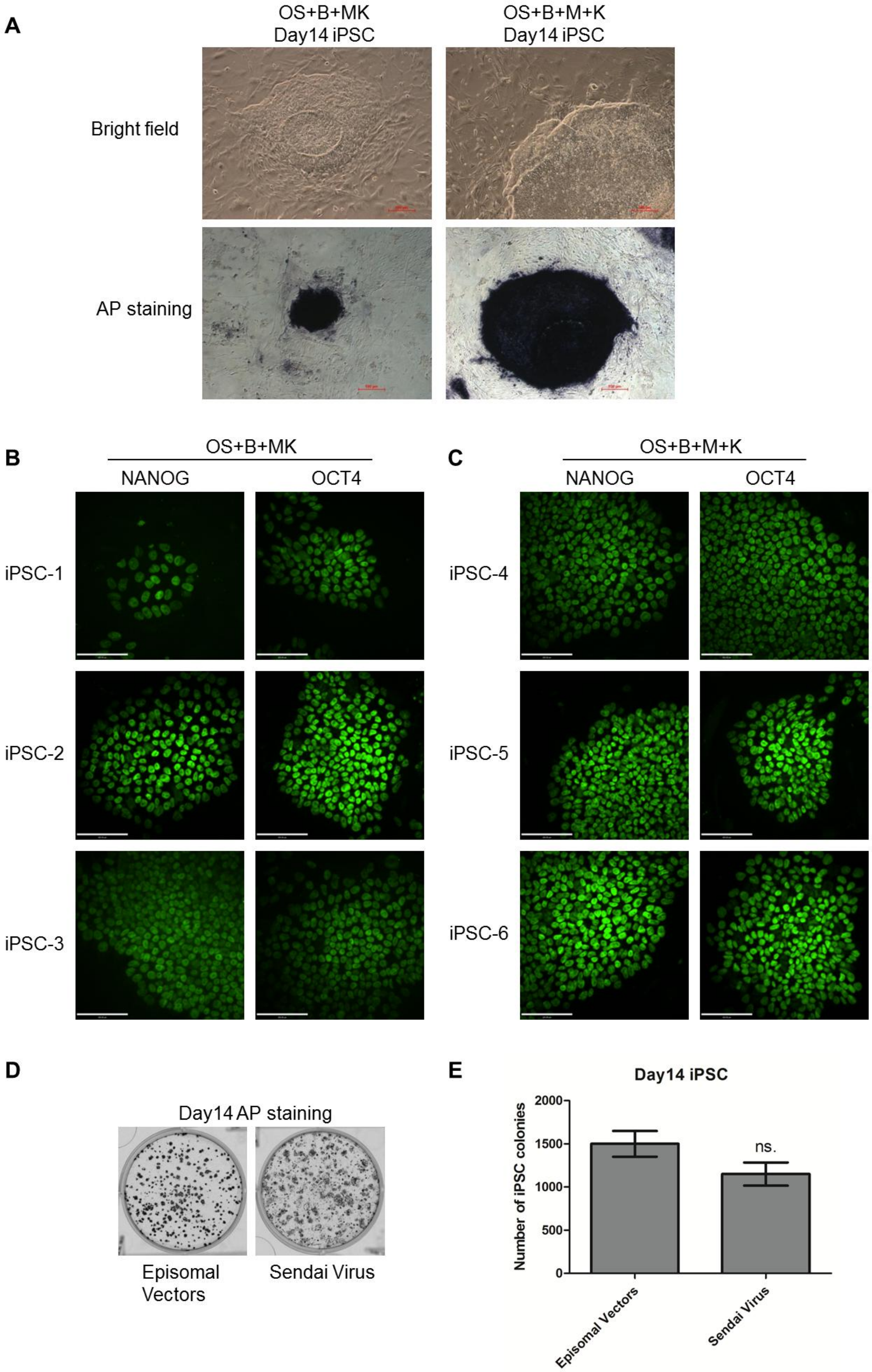

Figure S1 Related to Figure 1.

(A) Representative images of morphology and AP staining of iPSC colonies.

(B) and (C) Fluorescent images of iPSC colonies expressing OCT4 and NANOG. iPSCs were generated by nucleofection with OS+B+MK (B) and OS+B+M+K (C). Scale bar, 100 μm.

(D) Representative pictures of AP staining of iPSCs generated from 1×10^5 PB MNCs with the EV vs. Sendai virus reprogramming system.

(E) Numbers of iPSC colonies from 1×10^6 PB MNCs (Mean ± SEM, n = 3 independent experiments).

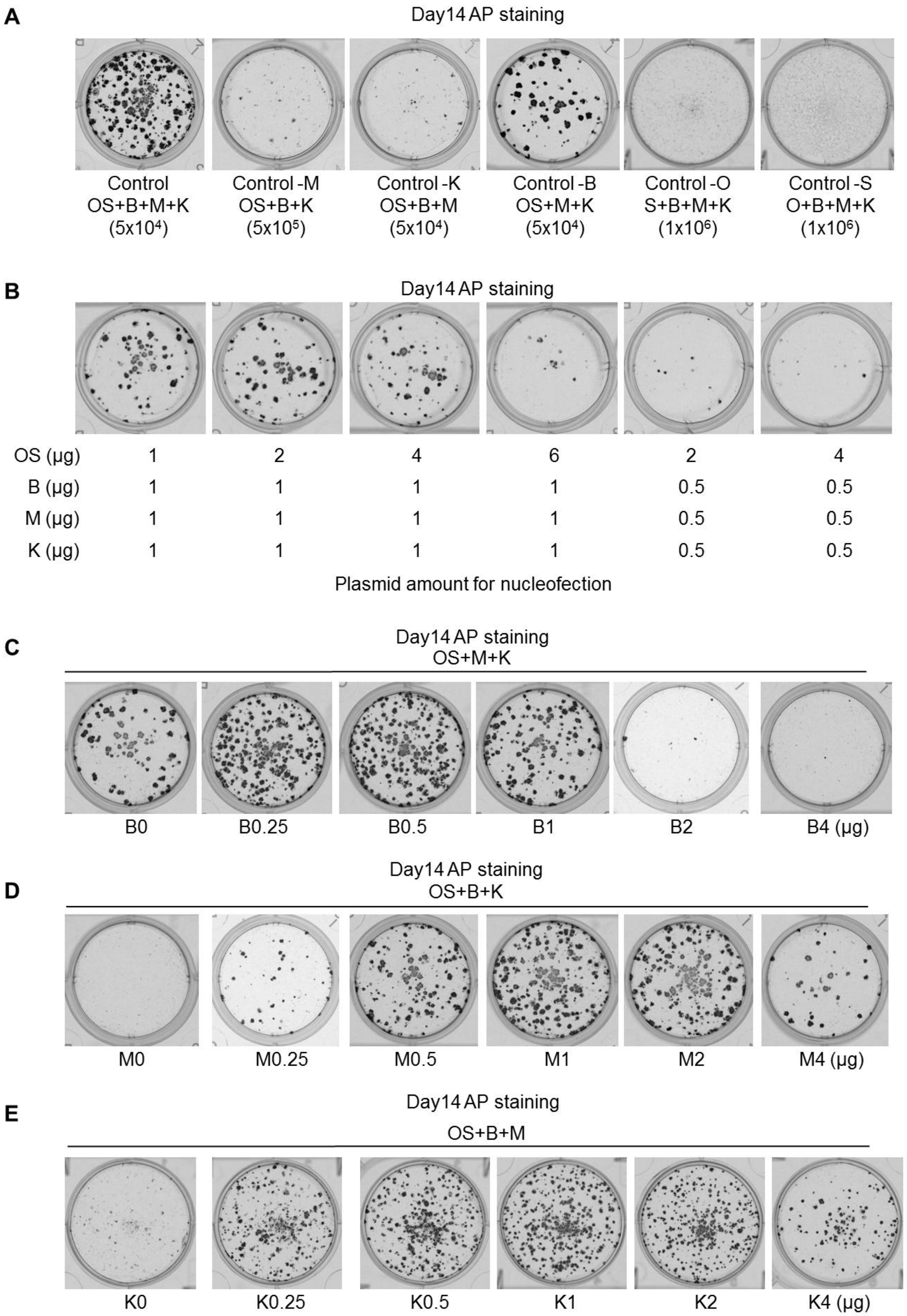
A
Day14 AP staining
Control
OS+B+M+K
(5x10⁴)
Control -M
OS+B+K
(5x10⁵)
Control -K
OS+B+M
(5x10⁴)
Control -B
OS+M+K
(5x10⁴)
Control -O
S+B+M+K
(1x10⁶)
Control -S
O+B+M+K
(1x10⁶)
B
Day14 AP staining
OS (µg) 1 2 4 6 2 4
B (µg) 1 1 1 1 0.5 0.5
M (µg) 1 1 1 1 0.5 0.5
K (µg) 1 1 1 1 0.5 0.5
Plasmid amount for nucleofection
C
Day14 AP staining
OS+M+K
B0
B0.25
B0.5
B1
B2
B4 (µg)
D
Day14 AP staining
OS+B+K
M0
M0.25
M0.5
M1
M2
M4 (µg)
E
Day14 AP staining
OS+B+M
K0
K0.25
K0.5
K1
K2
K4 (µg)

Figure S2 Related to Figure 2.

(A) Representative images of AP staining at day 14 post-transfection. Omission of one factor was tested in each condition. Note that the input cell numbers are different.

(B) Representative images of AP staining at day 14 post-transfection. $5x10^4$ PB MNCs were seeded in each well after nucleofection with indicated amount of each plasmid.

(C) Dosage effects of BCL-XL. Shown are representative images of AP staining of iPSCs 14 days after transfection with 2µg OS, 1µg M, 1µg K together with different amount of BCL-XL. $5x10^4$ PB MNCs were seeded in each well after nucleofection.

(D) Dosage effects of MYC. Shown are representative images of AP staining of iPSCs 14 days after transfection with 2µg OS, 1µg B, 1µg K together with different amount of MYC. $5x10^4$ PB MNCs were seeded in each well after nucleofection.

(E) Dosage effects of KLF4. Shown are representative images of AP staining of iPSCs 14 days after transfection with 2µg OS, 1µg B, 1µg M together with different amount of KLF4. $5x10^4$ PB MNCs were seeded in each well after nucleofection.

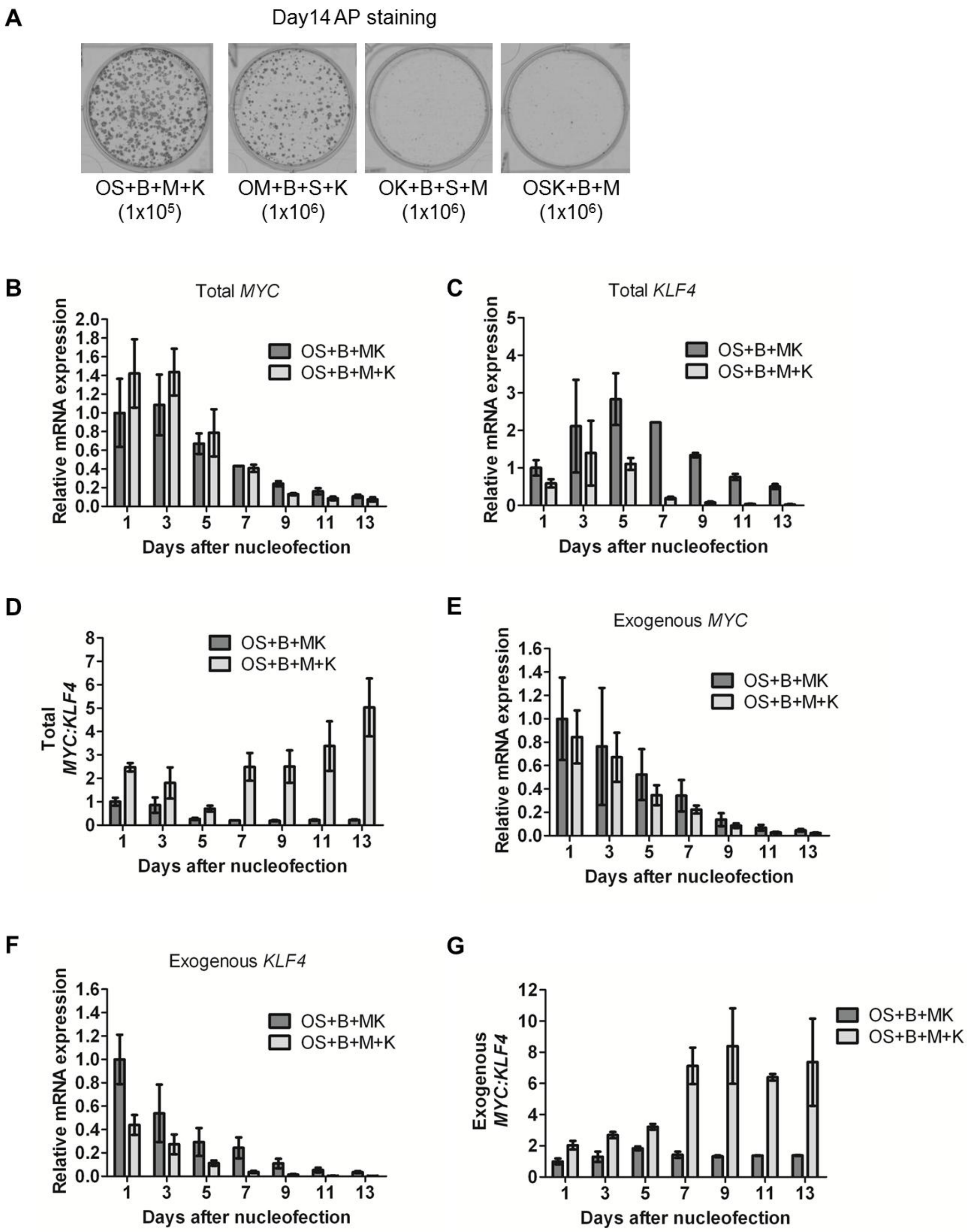
A
Day14 AP staining
OS+B+M+K (1x10^5)
OM+B+S+K (1x10^6)
OK+B+S+M (1x10^6)
OSK+B+M (1x10^6)
B
Total MYC
C
Total KLF4
D
Total MYC:KLF4
E
Exogenous MYC
F
Exogenous KLF4
G
Exogenous MYC:KLF4
Relative mRNA expression
Days after nucleofection
OS+B+MK
OS+B+M+K

Figure S3 Related to Figure 3.

(A) Representative images of AP staining of iPSC induction with different combinations of plasmids. Note that input cell numbers are different.

(B) Relative total *MYC* mRNA expression levels during reprogramming with OS+B+MK vs. OS+B+M+K. The expression levels are normalized to OS+B+MK at day 1 (Mean ± SEM, n = 3 independent experiments).

(C) Relative total *KLF4* mRNA expression levels during reprogramming in OS+B+MK vs. OS+B+M+K. The expression levels are normalized to OS+B+MK at day 1 (Mean ± SEM, n = 3 independent experiments).

(D) Relative ratios of total *MYC* to total *KLF4* during reprogramming in OS+B+MK vs. OS+B+M+K (Mean ± SEM, n = 3 independent experiments). The ratios are normalized to OS+B+MK at day 1.

(E) Relative expression of exogenous *MYC* at indicated time points after transfection with OS+B+MK vs. OS+B+M+K vectors. The expression levels are normalized to OS+B+MK at day 1 (Mean ± SEM, n = 3 independent experiments).

(F) Relative expression of exogenous *KLF4* at indicated time points after transfection with OS+B+MK vs. OS+B+M+K vectors. The expression levels are normalized to OS+B+MK at day 1 (Mean ± SEM, n = 3 independent experiments).

(G) Relative ratios of exogenous *MYC* to exogenous *KLF4* during reprogramming in OS+B+MK vs. OS+B+M+K (Mean ± SEM, n = 3 independent experiments). The ratios are normalized to OS+B+MK at day 1.

Supplemental Table

Table S1 Primer sequences used in the study.

Gene	Direction	Sequence
Total MYC	Forward	ACTCTGAGGAGGAACAAGAA
	Reverse	TGGAGACGTGGCACCTCTT
Exogenous MYC	Forward	TCCTCCGACAGACTGAGTC
	Reverse	CCTCCTCGTCGCAGTAGAAA
Total KLF4	Forward	TCTCAAGGCACACCTGCGAA
	Reverse	TAGTGCCTGGTCAGTTCATC
Exogenous KLF4	Forward	GAAATTCGCCCGCTCAGATG
	Reverse	TCATTTGCTGCCAGATCCTC
EBNA1	Forward	TTTAATACGATTGAGGGCGTCT
	Reverse	GGTTTTGAAGGATGCGATTAAG
Wpre	Forward	GGTTTAAACGCGTCGACAAT
	Reverse	GTTGCGTCAGCAAACACAGT
GAPDH	Forward	GAGTCCACTGGCGTCTTC
	Reverse	GACTGTGGTCATGAGTCCTTC

SUPPLEMENTAL EXPERIMENTAL PROCEDURES

AP Staining

The reprogramming efficiency was determined by counting AP-positive colonies at day 14. Cells were first fixed in 4% paraformaldehyde and subsequently stained for AP activities with an Alkaline Phosphatase Detection Kit (SiDanSai, China, 1102-100), following manufacturer's instructions. The colony numbers were scored by IQTL7.0 software or manually under a microscope.

Flow Cytometry

Cells were harvested with Accutase, followed by staining for 20 min at room temperature with PE-conjugated anti-TRA-1-60 (BioLegend; 330610), or eFluor 570-conjugated anti-SSEA4 (eBioscience; 41-8843). The cells were also stained with isotype antibody (eBioscience, 11-4011) for the negative control. After one wash with 2 ml PBS, cells were resuspend in 300 μl PBS for FACS analysis. Data were acquired using a BD LSR Fortessa cell analyzer.

Genomic DNA Extraction

Genomic DNA was extracted from cells using a Genomic DNA Extraction Kit (TIANGEN, DP304-02), following the manufacturers protocol.

Examination of residual plasmid by PCR

To determine the total copies of EV plasmid in iPSC lines, the Wpre and EBNA primers targeting the EV backbone were used (Table S1). PCR was performed using Phusion® High-Fidelity PCR Master Mix with HF Buffer Kit (BioLabs, M0531S). Briefly, 9 μl PCR grade water including 100 ng genomic DNA and 1 μl specific primers (10 μM each of forward and reward primers, Table S1) were added into 10 μl PCR Master Mix. The reaction mixture was incubated for 30 s at 98°C; followed by 10 s at 98°C, 30 s at 60°C and 30 s at 72°C for 35 cycles; and final extension for 5 min at 72°C. Genomic DNA from untransfected PB MNCs were used as a negative control. To quantify the EV copy number, 1.6 pg OS plasmid was mixed with 1 μg gDNA from untransfected PB MNCs to mimic cells with one copy of EV plasmid per cell.

RNA Extraction and cDNA Synthesis

Total RNA was extracted from cells using an RNeasy Mini Kit (Qiagen, 74104). cDNAs were synthesized with M-MLV Reverse Transcriptase (Invitrogen, 28025) and stored at -20°C until use.

Real-time PCR

Real-time PCR was performed using a FastStart Universal SYBR Green Master (Roche) on a Step One 7500 (Applied Biosystems). Briefly, 5 μl cDNA (1-10 ng) or 100 ng genomic DNA was added into 10 μl the SYBR Green Master together with 5 μl primers (2 μM each of forward and reward primers, Table S1). The reaction mixture was incubated for 10 min at 95°C, followed by 15 s at 95°C and 1 min at 60°C for 40 cycles. Data were analyzed using the Applied Biosystems Step One Software v2.2. Amplification of *GAPDH* was also conducted to control the quantity of loaded cDNA or gDNA in each reaction.

Confocal Imaging

iPSC lines were seeded onto feeder cells or Matrigel coated chamber slides. Cells were fixed in 4% paraformaldehyde for 30 min, followed by permeabilization treatment with 0.1% Triton X-100 in PBS for 30 min. After washing with PBS, cells were treated with blocking solution (5% goat serum in PBS, v:v) for 1 h, followed by overnight at 4°C with diluted primary antibody in blocking reagent. After two washes with permeabilization buffer, cells were incubated with a fluorophore-conjugated secondary antibody in blocking reagent for 2 h at room temperature. DAPI was used to stain the nucleus before imaging. Images were captured with a PE UltraVIEW confocal microscope.

Teratoma Assay

Approximately $1x10^6$ iPSCs after more than 5 passages in culture were harvested with Accutase and resuspended in 200 µl DMEM/F12 diluted (1:1) Matrigel solution (BD) and subcutaneously injected into the rear haunch of NOD/SCID immunodeficient mice. At 2 months after implantation, teratomas were dissected and fixed in 10% formalin. After microsectioning and staining with hematoxylin and eosin (H & E), samples were analyzed.

Karyotyping

iPSCs were cultured in Matrigel-treated 6-well plates. After reaching a 60%-70% confluence, cells were incubated with colchicines (0.2 µg/ml) for 3 to 4 hours, followed by treating with 0.05 mM EDTA for 3 min at 37°C. Cells were harvested with Accutase and single cell suspension was treated with 0.4% KCl for 25 min at 37°C. Cells were then fixed with 3-5 drops of fixation buffer (Methanol: Acetic Acid (v:v)= 3:1) for 5 min at room temperature. Finally, cells were centrifuged at 200 g for 8 min and treated with cold fixing buffer for 20 min at room temperature twice. The cell suspension was dropped onto cold clear slides followed by overnight incubation at 60°C in an incubator. The chromosomes were digested by 0.025% trypsin (Invitrogen) and stained with Giemsa stain solution (Sigma, 32884). The karyotyping analysis was performed using MetaSystem (Zeiss Imager Z2).
